# Single-immunocyte transcriptomics reveal the role of natural killer cell-dependent exogenous antigen presentation in ankylosing spondylitis severity

**DOI:** 10.1038/s12276-025-01619-6

**Published:** 2026-01-28

**Authors:** Dianshan Ke, Hanhao Dai, Yibin Su, Hongyi Zhu, Xiaofeng Liu, Xiaochun Bai, Changqing Zhang, Jie Xu, Jinshan Zhang

**Affiliations:** 1 Department of Orthopedics, Fuzhou University Affiliated Provincial Hospital, Fujian Provincial Hospital, Fuzhou, China; 2 Academy of Orthopedics, Guangdong Province, Guangdong Provincial Key Laboratory of Bone and Joint Degeneration Diseases, The Third Affiliated Hospital of Southern Medical University, Guangzhou, China; 3 Department of Orthopaedics, Shanghai Sixth People’s Hospital Fujian Hospital, Quanzhou, China; 4 Institute of Microsurgery on Extremities, Department of Orthopaedics, Shanghai Sixth People’s Hospital Affiliated to Shanghai Jiao Tong University School of Medicine, Shanghai, China; 5 State Key Laboratory of Organ Failure Research, Department of Cell Biology, School of Basic Medical Sciences, Southern Medical University, Guangzhou, China

**Keywords:** Ankylosing spondylitis, Autoimmunity, Cell biology, Medical genomics

## Abstract

Ankylosing spondylitis (AS) is an autoimmune disease that can cause severe deformities, and the immunological patterns associated with its onset and progression remain poorly understood. Here, after recruiting healthy donors and patients in different stages, we performed single-cell RNA sequencing for peripheral blood mononuclear cells to investigate the cytotaxonomic and immunological hallmarks associated with AS onset, aggravation and remission and explore the intrinsic laws causing AS lesions. The results showed that innate antibacterial defense functions were generally enhanced in most cell types at disease onset and were negatively associated with AS severity. The abundance and exogenous antigen presentation scores of the natural killer (NK) cell subset characterized as antigen-presenting cells (APC-NK) increased during disease aggravation but decreased during remission. Generally, APC-NK abundance and their presentation scores were negatively correlated with innate defense scores for multiple cell types. CD4^+^ effector T cell abundance and cytotoxicity, as well as the enhancement of CD4^+^ T cell responses by HLA-DRB1^+^ NK cells (similar to APC-NK), were associated with AS severity. The implantation of HLA-DRB1^+^ NK cells accelerated AS-like alterations in SKG modeling mice with curdlan induction; this was blocked with CD4^+^ T cell exhaustion. NK cell exhaustion improved the phenotypes of AS-like mice. HLA-DPB1/DPA1 in APC-NK participated in AS aggravation by mediating antigen presentation targeting CD4^+^ T cells. Overall, innate defense antigen presentation coupling drives AS lesions and different outcomes. Furthermore, the trade-off between innate defense and NK-dependent exogenous antigen presentation results in CD4^+^ T cell activation or inactivation, thereby contributing to AS aggravation or remission; this reveals that APC-NK is a crucial factor causing ankylosing deformities.

## Immune mechanisms drive ankylosing spondylitis progression

Ankylosing spondylitis (AS) is a chronic disease affecting the spine and joints, often leading to disability. Current treatments mainly focus on symptom relief, but understanding the disease’s underlying causes is crucial for better therapies. Researchers aimed to explore the immune processes involved in AS using advanced techniques such as single-cell RNA sequencing. The study involved 14 patients with AS and 3 healthy donors. Researchers collected blood samples and analyzed immune cells to understand their roles in AS. They found that certain immune cells, including T cells and natural killer cells, behave differently in patients with AS compared with healthy individuals. These differences are linked to the disease’s progression and severity. The study revealed that immune responses, particularly those involving NK cells, play a significant role in AS. These findings suggest that targeting specific immune pathways could improve treatment strategies.

This summary was initially drafted using artificial intelligence, then revised and fact-checked by the author.

## Introduction

Ankylosing spondylitis (AS) is a chronic rheumatic disease mainly damaging spine and peripheral joints^1,2^. Typical cases of AS can develop into spine and joint ankylosing deformities based on tissue fusion and destruction; these deformities ultimately result in disability^3,4^. Current therapies primarily focus on symptom relief and disease management, using drugs such as nonsteroidal anti-inflammatory drugs, conventional synthetic disease-modifying antirheumatic drugs, and biological DMARDs that inhibit tumor necrosis factor interleukin-17^5,6^. There are no other effective strategies besides surgical procedure to deal with late ankylosing destruction. Although targeted drugs have initiated research, they are not yet mainstream in treatment^5,6^. The improvement of treatment strategies relies on in-depth exploration of AS pathogenesis.

It is well known that patients with AS may achieve clinical remission after standard treatment at disease onset, but may progress to disability if untreated or if treatment is not standardized. Therefore, AS treatment regimens aim to rapidly induce remission, prevent disability and recurrence, and improve the condition of patients with established disability; achieving these goals requires a comprehensive understanding of the biological characteristics of AS at different stages, particularly its immunological hallmarks. The immunological mechanisms driving AS lesions have been depicted by many hypotheses that are based on HLA-B27^7–9^. However, the immunological processes causing AS onset and different outcomes still lack a clear explanation. Moreover, current studies on late ankylosing destruction mainly focus on aberrant osteogenesis^10–12^, and the relevant immunological laws have not yet made breakthroughs.

Single-cell RNA sequencing (scRNA-seq) based on immunocytes can help to improve our understanding at a deeper level. Previous scRNA-seq studies revealed some biological phenomena associated with AS^13–16^. However, the above studies were limited to comparisons between disease and healthy conditions and neglected the specific status of patients at different stages. The advantage of our study is that it integrates cytotaxonomic and immunological hallmarks in peripheral blood mononuclear cells (PBMCs) associated with AS onset (early active stage), aggravation (late dysfunction stage) and clinical remission, which contributes to the global understanding of pathogenic and prognostic factors in patients with AS and helps to explore the immunological laws associated with AS onset and outcomes.

## Materials and methods

### Patient recruitment and clinical characteristics

Three HLA-B27-positive healthy donors (HDs) with matching age were enrolled via genetic screening, and 14 patients with AS were recruited at Fujian Provincial Hospital and Shanghai Sixth People’s Hospital Fujian Hospital from May to July 2021. All patients with AS met the modified New York criteria, and the three disease groups (early active (EA), late dysfunction (LD) and clinical remission (RM)) were categorized on the basis of clinical hallmarks and imaging evidence. The five patients in the EA group were in the early stage of disease, showing obvious symptoms, and had not yet received standard treatments, including various nonsteroidal anti-inflammatory drugs and disease-modifying antirheumatic drugs. The five patients in the LD group were all disabled, confirmed by radiographic evidence of structural damage in hip joints and spinal deformity. The four patients in the RM group group received standardized treatment under the guidance of specialist physicians and maintained clinical remission and normal laboratory indicators for over 6 months, simultaneously meeting the following criteria: Bath Ankylosing Spondylitis Disease Activity Index (BASDAI) ≤3, C-reactive protein (CRP) ≤3.5 mg/l, and erythrocyte sedimentation rate (ESR) ≤15 mm/h. Patients in both EA and RM groups were confirmed to have no structural damage or deformities on the basis of radiographic evidence. With the approval of the Ethics Committee of Fujian Provincial Hospital (K2022-09-055) and informed patient consent, peripheral blood was collected from the three patient groups and three HDs for scRNA-seq. Tissue samples, including ligamentum flavum, posterior longitudinal ligament or interspinous ligament, were collected during surgeries from patients with AS (*n* = 5) and non-AS patients (*n* = 4). The additional nine HDs (HLA-B27-negative donors) and additional cases in the EA (eight), LD (seven) and RM (eight) groups underwent blood testing or bulk RNA-seq from 2021 to 2025. The clinical features of all individuals are presented in Supplementary Tables 1–3.

### Preparation of single-cell suspensions

The collected blood samples in the dedicated PBMC separation tubes (BD Vacutainer CPT Cell Preparation Tube, 362761, BD Biosciences) were centrifuged at 1500*g* for 30 min at 20 °C for extracting PBMCs from 14 patients and 3 HDs. Then, cells were washed twice with 1× phosphate-buffered saline (PBS) followed by erythrocyte lysis. Cells from each sample showed a viability rate over 90% and an aggregation rate under 5%.

### Cell capture and cDNA synthesis

Using the Single Cell 3′ Library and Gel Bead Kit v3.1 (10x Genomics, 1000121) and the Chromium Single Cell G Chip Kit (10x Genomics, 1000120), cell suspensions containing 300–600 live cells/µl (determined by Count Star) were loaded onto the Chromium Single Cell Controller (10x Genomics) to generate single-cell gel beads in emulsion according to the manufacturer’s protocols. The captured cells were lysed, and the released RNA was barcoded through reverse transcription in individual gel beads in emulsion. Reverse transcription was performed on a S1000 Touch Thermal Cycler (Bio-Rad) at 53 °C for 45 min, followed by 85 °C for 5 min, and then held at 4 °C. cDNA was generated, amplified and evaluated for quality using an Agilent 4200 (performed by CapitalBio Technology).

### scRNA-seq library preparation

According to the manufacturer’s instructions, scRNA-seq libraries were constructed using the Single Cell 3′ Library and Gel Bead Kit V3.1. The libraries were finally sequenced using an Illumina Novaseq6000 sequencer with a sequencing depth of at least 50,000 reads per cell with the pair-end 150 bp (PE150) reading strategy (performed by CapitalBio Technology).

### Single-cell raw data processing

Cellranger-6.0.1 software was obtained from https://pan.baidu.com/s/19Uixg202Zv4UdVL2vwthhg?pwd=18FP. Alignment, filtering, barcode counting and UMI counting were performed using the cellranger count module to generate a feature–barcode matrix and determine clusters. Dimensionality reduction was performed using principal component analysis, and the first ten principle compoments were used to generate clusters using a *K*-means algorithm and graph-based algorithm, respectively. Subsequently, FindNeighbors and FindClusters functions were used for cell clustering, and the RunUMAP function with default settings was used to perform nonlinear dimensional reduction.

### Multiple dataset integration

To compare cell-type dynamic changes across four groups, we used the integration methods described at https://satijalab.org/seurat/v4.0/integration.html44. The Seurat R package (v.4.0.0) was used to assemble multiple distinct scRNA-seq datasets into an integrated and unbatched dataset. In short, we identified 2000 features with high intercellular variability. Then, we used the FindIntegrationAnchors function to identify the ‘anchors’ between various datasets and inputted these anchors into the IntegrateData function to create a batch-corrected expression matrix for all cells, enabling the integration and analyses of cells from different datasets.

### Reclustering of T cells and NK cells

The five types of T cells in PBMCs were extracted and integrated for further reclustering. After integration, genes were scaled to unit variance. Principal component analysis and clustering were performed as described above. After extraction, natural killer (NK) cells were reclustered using the same procedure as for T cells.

### Annotation of cell types

After nonlinear dimensional reduction and projection of all cells into two-dimensional space through Uniform Manifold Approximation and Projection (UMAP), cells are clustered together on the basis of common features. FindAllMarkers function in Seurat was used to find the markers for each identified cluster. Then, clusters were merged/classified and annotated according to the expressions of typical markers for specific cell types. The intercellular communication was analyzed using CellChat (version 1.6.1) (https://github.com/sqjin/CellChat/issues/159).

### Identification and functional enrichment of DEGs

The expression of all intercluster differentially expressed genes (DEGs) were detected using FindMarkers function in Seurat with the parameter ‘test.use=wilcox’ by default, and the Benjamini–Hochberg method was applied to assess the false discovery rate (FDR). The acquisition of effective DEGs relies on gene filtering with a minimum log_2_(fold change) of 0.5 and a maximum FDR value of 0.01. The results were visualized using an R package. The Metascape network tool (www.metascape.org) was used to analyze the functional enrichments of DEGs. Datasets were obtained from the Gene Ontology (GO) database, specifically from the Biological Process category.

### Bulk RNA-seq of PBMCs

Total RNA was extracted from PBMCs obtained as described above and used to prepare libraries with the NEBNext Ultra RNA Library Prep Kit for Illumina (NEB). The fragmented mRNA template was synthesized into cDNA double strands, connected with sequencing adaptor and constructed into library using AMPure XP beads. The constructed library was initially quantified using Qubit2.0 Fluorometer, and the insert size was detected using Agilent 2100 bioanalyzer to ensure library quality. Different libraries were subjected to Illumina sequencing, and 150-bp paired-end reads were generated. After data quality control and sequence alignment analysis, read counts were normalized. Based on the differential expression analysis results, genes with a FDR ≤0.001 and log_2_(fold change, FC) ≥2 were considered significant DEGs for gene set variation analysis (GSVA).

### GSVA functional scorings

GSVA was used to evaluate functional enrichment scores between each group based on the gene expression in the gene sets. When analyzing individual gene sets, the preprocessed log_2_ gene expression matrix for each gene set was used as the GSVA input. GSVA runs separately on each gene set. Each signature requires a minimum of two genes.

### Single-cell trajectories analysis

After clustering UMAP coordinates, Slingshot v1.7.0 was used to fit a minimum spanning tree to B, pre-plasma and plasma B cells and determine the approximate trajectory structure. The segmented linear trajectory was smoothed using synchronous principal curves to obtain the final trajectory.

### Mendelian randomization (MR) analysis

Here, single-nucleotide polymorphisms (SNPs) were defined as instrumental variables (IVs). We obtained the overall data on AS from FinnGen Consortium, with gwasID: finngen_R12_M13_ANKYLOSPON (https://r12.finngen.fi/pheno/M13_ANKYLOSPON), including 3838 cases and 353,224 controls, with approximately 20,110,731 SNPs. We obtained the overall data on pneumonia from IEU open gwas, including 16,887 cases and 463,412 controls, with 24,174,646 SNPs. We selected SNPs with *P* ≤ 5 × 10^-6^ as the significance threshold, and used the link-disequilibrium aggregation process (*r*^2^ = 0.001, kilobases (kb) = 10,000) to exclude relevant SNPs, thereby ensuring the independence of the selected IVs. Then, the *F*-statistic of these IVs was calculated to evaluate their strength. SNPs with an *F*-statistic less than 10 were excluded to eliminate weak tool bias. Using IVW as the main tool, we evaluated the causal relationship between pneumonia and AS risk. Weighted median, MR-Egger, simple model and weighted model were also used as supplementary methods for IVW. The threshold for statistical differences is *P* < 0.05. The software used in this study is RStudio (4.3.1), and the main R package is TwoSampleMR (0.5.7). The data used are publicly available and approved by institutional review committees in the respective studies. Therefore, no further approval is required. This study used pneumonia as the exposure and AS as the outcome. There is a strong correlation between SNPs and exposure factors; SNPs are independent of confounding factors, and SNPs can only affect outcomes through exposure factors.

### Cell enrichments through fluorescence-activated cell sorting (FACS)

After blockage of nonspecific binding using Fc Receptor Blocking Solution (Human TruStain FcX, BioLegend), cells were resuspended in Dulbecco’s PBS supplemented with 10% bovine serum albumin and stained for CD3-FITC (317306, BioLegend), CD3-PE (317308, BioLegend), CD4-APC (300514, BioLegend), GZMB-PerCP (396416, BioLegend), CD8a-PE (300908, BioLegend), KLRF1/NKp80-APC (346708, BioLegend), HLA-DRB1-PE (362301, BioLegend), CD4-APC (100412, BioLegend), CD3-PerCP/Cyanine5.5 (100217, BioLegend) and NK1.1-APC (156505, BioLegend) antibodies according to different sorting strategies. The sorted cells were used for subsequent experiments.

### Flow cytometric analysis

For flow cytometric analysis, we used CD3-PE, KLRF1/NKp80-APC, CD19-FITC (363008, BioLegend), CD56-FITC (362546, BioLegend), GZMB-PE (372207, BioLegend), GZMB-FITC (372205, BioLegend), CD4-APC (300514/100412, BioLegend), CD4-PerCP (344623, BioLegend), NK1.1-APC (156505, BioLegend), HLA-DRB1-PE (362301, BioLegend), IFN-γ-PE (383403, BioLegend), KLRK1-PE (206303, BioLegend) and HLA-DPB1-PE (bs-4107R, Bioss) antibodies for staining by standard protocols. In addition, the apoptosis levels in enriched mouse NK cells or CD4^+^ T cells was evaluated using Annexin V–fluorescein isothiocyanate and propidium iodide staining via standard protocols.

### Quantitative real-time PCR assays

The total RNA was extracted and purified using TRIzol methods. cDNA synthesis and real-time quantitative PCR assays were performed according to the manufacturer’s protocols (Takara). The predesigned primer sequences are presented in Supplementary Table 4. After reaction, the amplification curve and melting curve were confirmed. Each melting curve showed a single peak at 80–90 °C, and the peak shapes were narrow. No primer dimer curve peak or other heteropeaks were found. Accordingly, the specificity of each primer was qualified. The cycle threshold (C_t_) value was calculated using the housekeeping gene GAPDH as the internal control. The relative expression of target genes in experimental samples was determined using control samples as the reference.

### Lentiviral infection

Lentiviral vectors encoding human DPB1-cDNA, DPA1-cDNA, DPB1-shRNA and DPA1-shRNA were purchased commercially (cDNA: pRRLSIN-cPPT-SFFV-MCS-3FLAG-E2A-EGFP-SV40-puro; shRNA: pRRLSIN-cPPT-U6-shRNA-SFFV-EGFP-SV40-puro; GeneChem). NK cells under fine condition were seeded onto 96-well plates and incubated at 37 °C in 5% CO_2_ overnight. Cells (1.2 × 10^4^ cells per well) were infected for 24 h within the viruses in the presence of polybrene (5 μg/ml). Then, original medium was replaced by the medium containing fresh fetal bovine serum. Three days later, antibiotic selection was conducted by adding puromycin (1.0 μg/ml) into medium during each replacement, for 7 days before expansion. The transduction efficiency was validated by western blotting.

### Western blot assays

Cells were ultrasonically treated into lysis buffer with phosphatase and protease inhibitors (KGP2100, Keygen Biotech). Cell lysates were transferred onto polyvinylidene fluoride (PVDF) membranes. After blockage with 5% milk, PVDF membranes were incubated with GAPDH (1:25,000, 10494-1-AP, Proteintech), HLA-DPB1 (1:2000, bs-4107R) and HLA-DPA1 (1:1000, bsm-60290R, Bioss) antibodies at 4 °C overnight. Subsequently, PVDF membranes were incubated with HRP-conjugated goat anti-rabbit secondary antibody (SA00001-2, Proteintech) at 37 °C for 1 h. The signals were visualized using an Omni-ECL Enhanced Pico Light Chemiluminescence Kit (SQ101, Epizyme Biomedical Technology) and automatic digital gel/chemiluminescence image analysis system (4600SF, Tanon).

### Coculture of CD4^+^ T cells with corresponding NK cells and subsequent assays

Twenty-four hours before coculture, CD4^+^ T cells were seeded on 96-well plates coated with CD3 and CD28 antibodies (2C11, 37.51; BD Pharmingen) at 1 × 10^4^ per well in proliferation medium (RPMI supplemented with 10% fetal bovine serum, 5 × 10^−5^ M β-mercaptoethanol and sodium pyruvate), and stained with 5 µM carboxyfluorescein-succinimidyl-ester (CFSE; Elabscience). After FACS enrichment, HLA-DRB1^+^ NK cells from different groups of participants at a ratio of 1:1 or HLA-DPB1/DPA1-overexpressed NK cells at a ratio of 3:1 were added to T cells in the presence of 20 μg/ml ovalbumin peptides (A8040, Solarbio) at 37 °C for 48 h. For T cell proliferation assays, CD4^+^ T cell proliferation was evaluated by flow cytometry as indicated by dilution of CFSE fluorescence. The detection of CD4^+^ T cytotoxicity relied on flow cytometry analyses of CD4-PerCP and GZMB-FITC antibodies or flow cytometry analyses of GZMB-FITC/PE antibodies in CD4^+^ cells sorted by FACS. Without permeation, the contacts between HLA-DPB1/DPA1 and CD4 on the surface of CD4^+^ T cells in the coculture medium were observed by flow cytometry (EGFP and PerCP fluorescence). Co-immunoprecipitation assay was carried out using anti-CD4 magnetic beads immunoprecipitation (IP) kit (MB50134-T52, Sino Biological) via standard protocols. In brief, indicated cells were lysed using IP lysis buffer (PC105, Epizyme), and IP was conducted by incubation with magnetic beads overnight at 4 °C. The beads were rinsed three times with lysis buffer. The precipitates were eluted with loading buffer (LT101, Epizyme) and isolated for western blot analyses with Flag (1:10,000, bsm-33346M, Bioss), HLA-DPB1 (1:1000, bs-4107R), HLA-DPA1 (1:500, bsm-60290R) and CD4 (1:5000, ab133616, Abcam) antibodies.

Next, we searched for ovalbumin peptide with potential to strongly bind to DPB1/DPA1 through the MHC-II Binding Prediction website (http://tools.iedb.org/mhcii/). After entering the protein sequence of ovalbumin, we chose NetMHCIIPan 4.1 EL as the prediction method, and the allele range of HLA-DP spanned from DPA1*01:03/DPB1*01:01 to DPA1*01:03/DPB1*20:01. The sequence length ranges from 12 to 18. According to the search results, OVA127-141 exhibits percentile RANK of less than 0.3 across 10 alleles of DPB1/DPA1 (Supplementary Table 5), indicating strong binding affinity (%Rank <2% indicates strong MHC binder). A DNA coding sequence corresponding to the OVA127-141 amino acid sequence was designed (AGAGGAGGCTTGGAACCTATCAACTTTCAAACAGCTGCAGAT), and the vector carrying OVA127-141 cDNA (pcDNA3.1) was constructed through Consure Bio. NK cells were incubated in opti-MEM with transfection complexes containing OVA127-141 cDNA using LipoBooster 3000. After 48 h of transfection, the overexpression efficiency of OVA127-141 was evaluated using a specific antibody (GenScript Biotech). OVA127-141-overexpressed NK cells at a ratio of 3:1 were added to the CD4^+^ T cells at 37 °C for 48 h, and then OVA127-141 expression in sorted CD4^+^ cells was detected using OVA127-141-specific antibody.

### Induction, treatment and assessment of AS modeling mice

Female SKG mice were purchased from GENEANDPEACE and raised at the Laboratory Animal Center of Shanghai Sixth People’s Hospital Fujian Hospital under specific pathogen-free conditions. They underwent 12-h light/dark cycling in a temperature (22 ± 2 °C)- and humidity (60%)-controlled room with free access to water and food. All mice were treated according to the guidelines for animal care approved by the Institutional Animal Care and Use Committee of Fujian Provincial Hospital (K2022-09-055). AS-like alterations were induced at 10 weeks of age using 3 mg curdlan (Wako Chemicals) by intraperitoneal injection. SKG modeling mice were randomly divided into six groups (*n* = 5 per group): (1) PBS + CD4-promoter-Cont-AAVs group; (2) PBS + CD4-promoter-DTR-AAVs group; (3) DRB1^+^ NK-cells+CD4-promoter-Cont-AAVs group; (4) DRB1^+^ NK-cells+CD4-promoter-DTR-AAVs group; (5) PBS + NK1.1-promoter-Cont-AAVs group; (6) PBS + NK1.1-promoter-DTR-AAVs group; (7) DRB1^+^ NK-cells+NK1.1-promoter-DTR-AAVs group; (8) DRB1^−^ NK-cells+NK1.1-promoter-DTR-AAVs group. The AAV9 vectors with NK-cell-specific NK1.1-promoter or CD4^+^ T-cell-specific CD4-promoter carrying DTR-cDNA or control plasmids were purchased commercially (GeneChem), and adeno-associated viruses (AAVs) were diluted to 1 × 10^13 ^μg/ml using PBS. SKG modeling mice in the first four groups were treated with 100 μl AAVs via tail-vein injection after 1 week of induction along with PBS or enriched HLA-DRB1^+^ NK cells from LDs (3 × 10^4^), and administered intraperitoneally with 400 ng diphtheria toxin (DT) 2 weeks later and continued to receive 2 weeks of induction. SKG modeling mice in the last four groups were injected with 100 μl corresponding AAVs after 1 week of induction along with enriched HLA-DRB1^+^ or HLA-DRB1^−^ NK cells from LDs (3 × 10^4^) and administered intraperitoneally with 400 ng DT 2 weeks later and continued to receive 5 weeks of induction. The two independent observers who were blinded to grouping monitored the clinical characteristics in the mice weekly. The score criteria are as mentioned previously^17^: 0 = no swelling or redness, 0.1 = swelling or redness of the digits, 0.5 = mild swelling and/or redness of the wrist or ankle joints, and 1 = severe swelling of the larger joints. After 5 or 8 weeks of induction, all mice were euthanized and the tissues were obtained for micro-computed tomography (micro-CT) scannings, hematoxylin and eosin (H&E) staining and Safranin-O/Fast Green (SO-FG) staining as described below. The remaining mice were randomly divided into four groups (*n* = 3 per group) and only received AAV administration or HLA-DRB1^+^ NK cell implantation, and flow cytometric analysis or apoptotic assessment was conducted 10 days after diphtheria-toxin injection (absent in cell assays with equivalent intervention time); this was used to identify the effectiveness of the aforementioned AAVs and the in vivo role of APC-NK on CD4^+^ T cell activation.

### Micro-CT scanning

Micro-CT analysis was performed on the structures of mouse ankle and spine samples. The collected tissues were fixed with 4% paraformaldehyde and then scanned with a SkyScan1276 CT scanner (version 1.5) with a resolution of 6 μm (mouse ankle joint) or 10 μm (mouse spine). The figure data were analyzed using CTAn analysis software (version 1.20.3.0).

### Histological assessment

The positions of ossified ligaments in patients with AS were first confirmed through the preoperation imaging examinations and visual observations during surgery. Human ligament tissues and mouse ankle and spine samples were fixed with 4% paraformaldehyde for 24 h, decalcified in 20% EDTA for 14 days and then embedded in paraffin for preparing sections. The indicated sections were stained with hematoxylin for 5 min. After rinsing with PBS, eosin staining was performed for 5 min. Then, the dehydrated sections were observed and photographed using a microscopy. SO-FG staining was also performed to assess the above samples. The corresponding tissues were fixed, decalcified, embedded and sectioned as described above, and then stained with 0.02% Fast Green FCF and 0.1% Safranin O. Finally, the sections were observed and photographed using a microscopy. For immunofluorescent staining, the sections were deparaffinized, hydrated and incubated in 1% Triton X-100. After incubating in 10 mM citrate buffer (pH 6.0) at 60 °C overnight to disclose antigens, the sections were incubated with CD4 (1:250, sc-19641, Santa Cruz Biotechnology) and HLA-DRB1 (1:100, bsm-63257R, Bioss) antibodies overnight at 4 °C and then incubated with fluorescein-conjugated secondary antibody for 1 h. Finally, 4′,6-diamidino-2-phenylindole was added to sections for 15 min to counterstain. All images were obtained using an inverted fluorescence microscopy (Leica DMI8). CD4^+^ cells and double-positive cells expressing both CD4 and HLA-DRB1 were quantified using ImagePro Plus (IPP) 6.0 software. Under high magnification, three areas were selected and positive cells were counted to obtain the overall positivity rate. The sections were randomly selected and scored by two blinded observers.

### ELISA assays

Serum levels of IFN‑λ1 (EK41703), IFN‑λ2 (EK3877), and IFN‑λ3 (EK4203) were measured using enzyme-linked immunosorbent assay (ELISA) kits (Signalway Antibody) according to the manufacturer’s protocols.

### Statistical analysis

Independent one-way or two-way analysis of variance (ANOVA) and Student’s *t*-tests were applied to analyze the data after determining their normal distribution. Tukey’s test was used for post-hoc multiple comparisons of ANOVA. All experiments were carried out using at least three independent samples. Data are represented as the mean ± s.d. The threshold for *P* values is set to 0.05. All statistical analyses were performed using IBM SPSS26.0 software (IBM Corp.). ggcorrplot2.R (https://github.com/caijun/ggcorrplot2) was used for correlation analysis between various parameters of patients.

## Results

### Single-immunocyte transcriptional profiling

**Fig. 1:**
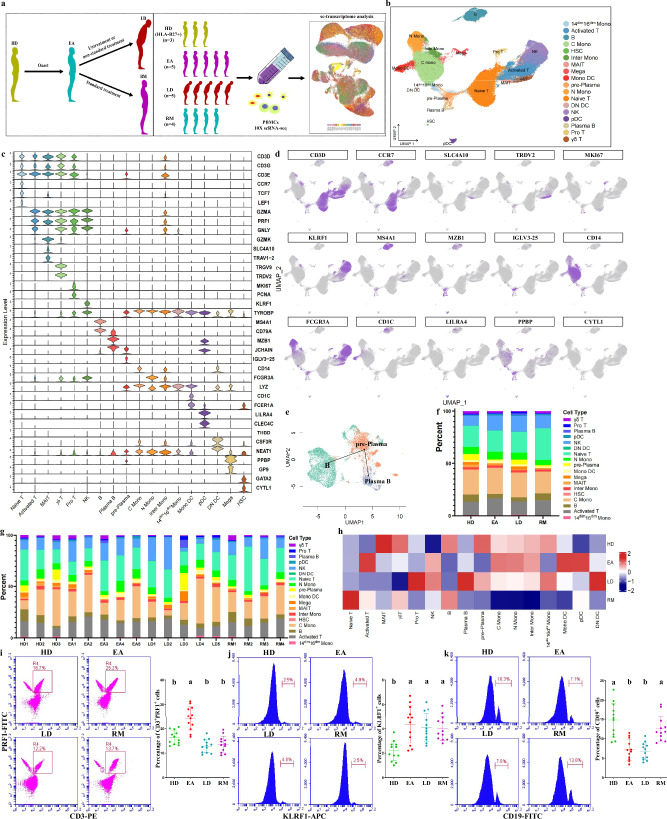
Study design and single-immunocyte transcriptional profiling. **a** The overall study design showing that, after AS onset, patients in the early stage develop into patients in late stage due to untreatment or nonstandard treatment, or obtain clinical remission following standard treatment. The scRNA-seq was based on PBMCs across four conditions, and the output data were applied for single-cell transcriptome analysis. **b** UMAP of 118,334 single cells from HLA-B27^+^ HDs (*n* = 3), EAs *(n* = 5), LDs (*n* = 5) and RMs (*n* = 4) showing 18 annotated cell types with representative labels. Each dot corresponds to a single cell and is colored according to cell type. **c** Violin chart showing the expression distribution of typical cell markers in 18 cell types. The rows indicate various markers, and the columns indicate various cell types. **d** Typical cell markers in individual UMAP plots were colored according to the expression levels and distributions. **e** Slingshot trajectory analysis of B cells and two effector B cells revealing a dominantly linear trajectory according to high similarity between two lineages. **f** Proportions of various cell types from four groups. **g** Proportions of various cell types at sample levels. **h** Heat map analysis for intergroup comparison regarding the proportions of 16 main cell types. Columns were normalized, and the transition of blue–white–red indicates an increase in the proportion. **i**–**k** Flow cytometry identifying the approximate trend of activated T cells (CD3 and PRF1 antibodies) (**i**), NK cells (KLRF1 antibody) (**j**) and B cells (CD19 antibody) (**k**) calculated from scRNA-seq. Data were obtained from PBMCs of 12 participants in each of the HD, EA, LD and RM groups. Different letters indicate statistically significant decreases with *P* < 0.05. In **i**–**k** one-way ANOVA and Tukey’s post-hoc multiple comparisons were used.

First, we conducted a scRNA-seq to investigate the transcriptomic characteristics of PBMCs from 14 patients and 3 HDs (Fig. 1a and Supplementary Table 1). Patients with AS were divided into three groups based on respective clinical characteristics: initial patients with high activity (EAs); patients with spinal deformities or joint destruction (LDs); and patients with clinical remission (RMs) (Fig. 1a). High-quality cells of each group were subjected to integration after correcting read depth and mitochondrial read count (Supplementary Fig. 1a,b). Relying on Seurat-based clustering of UMAP, we captured transcripts of 18 major cell types as following: naive-state T (naive T), activated-state T (activated T), mucosa-associated invariant T (MAIT), γδ T, proliferative T (pro T), NK, B, plasma B, pre-plasma, classic monocytes (C monos), nonclassic monocytes (N monos), intermediate monocytes (inter monos), monocytes with low expression of *CD14* and *CD16* (14^dim^16^dim^ monos)^18^, monocyte-derived dendritic cells (mono DCs), plasmacytoid DCs (pDCs), *CD1C*^-^*CD141*^−^ DCs (DN DCs)^19^, megacaryocytes (megas) and hemopoietic stem cells (HSCs) (Fig. 1b–d and Supplementary Table 6). The data highlighted a cluster of cells with low *MZB1* expression, high *JCHAIN* expression and marked expression of multiple IGV genes; these cells were defined as pre-plasma cells on the basis of their genetic characteristics and adjacency to plasma B cells (Fig. 1b–d and Supplementary Table 6). Slingshot-trajectory analysis also revealed that pre-plasma cells were in a transitional position (Fig. 1e), which was also confirmed by the strongest clonal sharing and connectivity between pre-plasma and plasma B cells (Supplementary Fig. 1c,d).

Intergroup comparison of cell types showed that, compared with other groups, the proportion of naive T cells in RMs sharply increased (Fig. 1f–h). The activated T cell proportion in EAs or pro T cell proportion in LDs was the highest among the four groups, respectively (Fig. 1f–h). NK cell proportions in AS groups all increased (Fig. 1f–h). Activated T cell and NK cell trends were identified by flow cytometry showing that CD3^+^PRF1^+^ cells were the most abundant in EAs and KLRF1^+^ cells increased in three AS groups (Fig. 1i,j). Compared with HDs or RMs, B cell proportions in EAs and LDs were lower (Fig. 1f–h). Plasma B cell proportions increased progressively from HDs to LDs, but were decreased in RMs compared with EAs (Fig. 1f–h and Supplementary Fig. 1e). Pre-plasma cell proportions decreased in EAs but sharply increased in LDs (Fig. 1f–h and Supplementary Fig. 1e). Therefore, we suggest that both AS onset and aggravation are characterized by B cell rapid maturation whereas AS remission exhibits delayed B cell development; this was supported by the observation that CD19^+^ cells were more abundant in HDs/RMs than in EAs/LDs (Fig. 1k). In addition, AS onset displays obvious immune-activating characteristics, as indicated by the increased abundance of three effector-lymphocyte types (activated T, plasma B and NK), in accordance with previous studies showing that the development of immature cells into effector lymphocytes is the basic pathology of autoimmune diseases^20,21^ and revealing the role of effector lymphocytes in AS/spondyloarthritis (SpA)^6,22–25^. These results suggested that some immunosuppressants may possess positive efficacy at an early stage.

### Functional changes in immunocytes across AS conditions

**Fig. 2:**
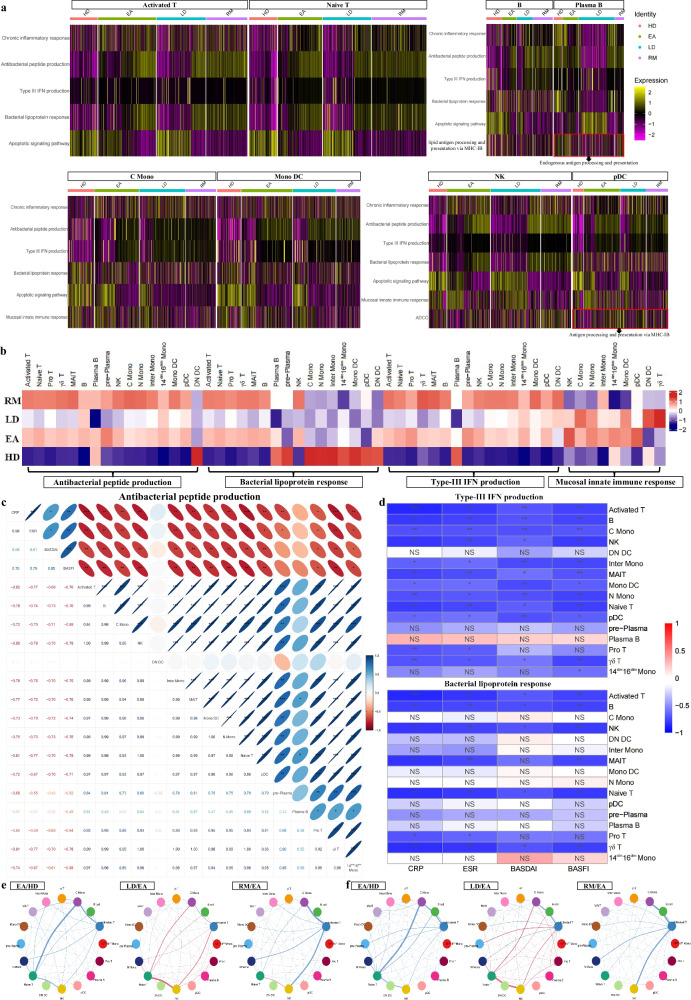
Functional changes in immunocytes across AS conditions. **a** Heat maps showing relative intergroup comparisons of single-cell GSVA scores in multiple functions from corresponding cell types (lipid antigen processing and presentation via MHC-IB and antigen processing and presentation via MHC-IB are different forms of endogenous antigen presentation). Rows were normalized, and the transition of purple–black–yellow indicates an increase in the scores. **b** Relative intergroup comparisons of overall GSVA scores for specific functions of various cell types. Columns were normalized, and the transition of blue–white–red indicates an increase in the scores. **c**,**d** Correlation analysis between innate defense function scores (antibacterial peptide production, bacterial lipoprotein response and type-III IFN production) of 16 main cell types and various clinical parameters (CRP, ESR, BASDAI and BASFI) from patients. The number of asterisks indicates the FDR-adjusted *P* value (*q* value) (*q < 0.05, **q < 0.01, ***q < 0.001), and different colors indicate the correlation coefficient (*R* value). NS, not significant. **e** Intercellular communications showing differential action strength from five types of T cells to other cell types between corresponding groups. **f** Intercellular communications showing differential action strength from other cell types to T cell types between corresponding groups.

We relied on GSVA to explore key functional changes. Chronic inflammatory response in most cell types was stronger in AS groups than in HDs, and that in EAs was the strongest for most cell types (Fig. 2a,b and Supplementary Fig. 2a). Antibacterial peptide production in EAs was stronger than in HDs or LDs and weaker than in RMs for most cell types (Fig. 2a,b and Supplementary Fig. 2a). Bacterial lipoprotein response in EAs was stronger than in HDs or LDs and weaker than in RMs for five T cell types, B cells and NK cells (Fig. 2a,b and Supplementary Fig. 2a). These results are consistent with AS pathogenesis, which is characterized by autoimmune inflammation triggered by multiple bacterial infections^26,27^. In addition, type-III interferon (IFN) production in AS groups was stronger than in HDs for most cell types, and compared with EAs, LDs had lower values and RMs had higher values for most cell types (Fig. 2a,b and Supplementary Fig. 2a). ELISA results confirmed these trends, showing that serum IFN‑λ1 levels in AS groups were significantly higher than in HDs, with LDs showing the lowest levels and RMs showing the highest within the AS groups (Supplementary Fig. 2b). Type-III IFN with high expression in patients with AS is induced by bacterial ligands and restricts bacterial infection^28,29^. The mucosal innate immune response in EAs was stronger than in HDs or RMs for most innate immunocyte types but was overall unaltered compared with LDs (Fig. 2a,b and Supplementary Fig. 2a). Therefore, AS onset is associated with strong mucosal innate immunity—the first line of defense against pathogen invasion—which has been shown to play a key role in SpA pathogenesis^30^. By integrating multiple AS-triggering factors, our data not only provide a detailed interpretation for the characteristics reported in previous studies but also uncover new factors associated with AS, suggesting that infection-induced mucosal innate immunity via type-III IFN is the driving factor for AS lesions. As an antibiotic drug, sulfasalazine is considered an important conventional synthetic disease-modifying antirheumatic drug with significant efficacy in treating axial and peripheral AS^5,31^, and its relevance was further supported by scRNA-seq analysis. However, AS aggravation was characterized by the reduction in most functions whereas AS remission exhibited optimal antibacterial properties accompanied by weak inflammation and mucosal innate immunity. These results provide insights into achieving and maintaining a mitigatory state by highlighting the role of antibacterial activity in reducing immune inflammation. This is further supported by MR analyses, which indicate that infection serves as a risk factor for AS (Supplementary Fig. 3a–d).

Importantly, the above results depict an outline for innate antibacterial defense: bacteria act on immunocytes via mediators such as lipoprotein, driving the bacteriostatic effect of immunocytes, including the production of antibacterial peptides and type-III IFNs.“Integration of multiple datasets revealed a meaningful biological phenomenon: innate defense is enhanced at AS onset but varies inversely with disease severity. This was supported by bulk RNA-seq of multiple PBMC samples, which showed that GSVA scores for antibacterial peptide production and type-III IFN production were increased in EAs, while three innate defense functions were negatively associated with disease severity (Supplementary Fig. 2c). These observations indicate that innate defense may be a favorable factor for improving conditions. Similar to the observed trend for innate defense, antibody-dependent cellular cytotoxicity (ADCC) in N monos and NK cells from EAs was stronger than that from HDs but weaker than that from RMs, and ADCC of LDs was weaker than ADCC of EAs in N monos (Fig. 2a and Supplementary Fig. 2a). The significance of ADCC to innate defense also supports the protective effect of innate defense on patients with AS. As expected, the scores for three innate defense functions in most cell types were negatively correlated with clinical parameters including CRP, ESR, BASDAI and the Bath AS Function Index (BASFI) in patients (Fig. 2c,d). Moreover, endogenous antigen processing and presentation was the strongest in EAs for B and plasma B cells, and that for pDCs was associated with AS severity (Fig. 2a). The simultaneous increase in endogenous antigen presentation, antibacterial peptide production and type-III IFN production for three classic antigen-presenting cells (APCs) in EAs implied the contribution of infection-induced endogenous antigen presentation to AS onset. This not only reinforces the role of abnormal peptide processing in driving AS lesions but also provides additional theoretical support for the hypothesis linking HLA-B27 to AS^9,32,33^.

Intercellular communications displayed that, based on EAs, T cells showed an overall increased signal output in LDs while showing an overall decreased output in RMs (Fig. 2e), revealing that T-cell-mediated signal transmission is associated with AS severity. T cells use secreted cytokines to influence other immune cells, suggesting that therapeutic improvement could be achieved by inhibiting T cell cytokine production. In addition, signal transmission from other immunocytes to T cells was presented in pDCs and plasma B cells from EAs, but based on EAs, various T cells showed an overall increased signal input in LDs while showing an overall decreased input in RMs (Fig. 2f and Supplementary Fig. 2d), revealing that T-cell-received signal transmission also participates in AS onset and outcomes. The reverse communication of multiple APCs to T cells indicates that antigen presentation is a key factor driving AS onset and progression, and its attenuation is critical for disease improvement.

### NK cell dynamics across AS conditions

**Fig. 3:**
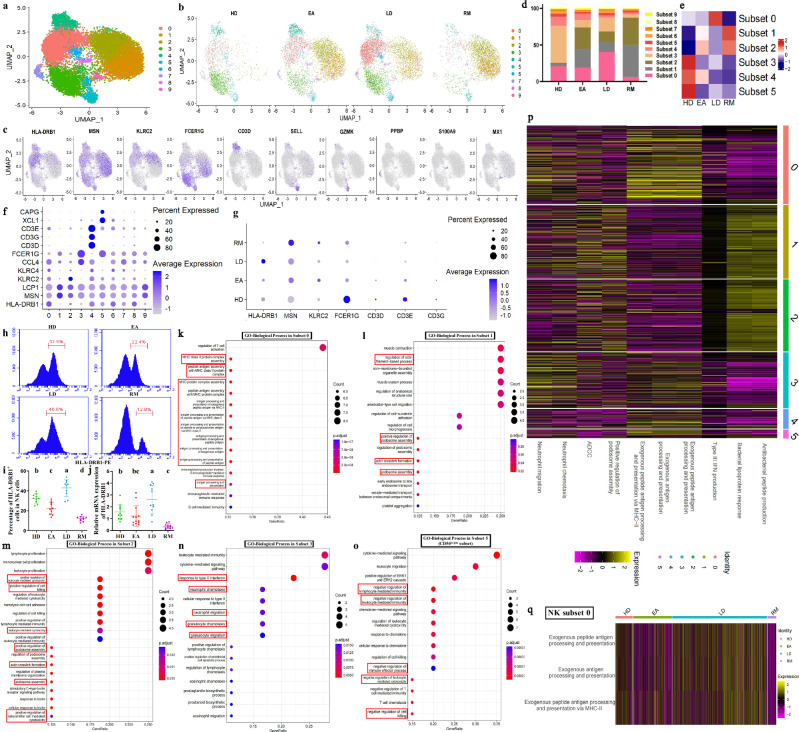
NK cell dynamic features across AS conditions. **a** UMAP projection of 15,464 NK cells. Each dot corresponds to a NK cell and is colored according to cell cluster. **b** UMAP projection of four conditions. **c** Typical markers in individual UMAP plots were colored according to the expression distribution. **d** Proportions of ten NK subsets from four groups. **e** Intergroup preferences of NK subsets 0–5. Rows were normalized, and the transition of blue–white–red indicates an increase in the proportion. **f** Bubble plots showing the expression distribution of top genes for subsets 0–5. **g** The expression distribution of top genes for subsets 0–4 across four groups. Circle size represents the percentage of cells expressing the corresponding gene (PCT), and color intensity represents the average expression level (**f** and **g**). **h**,**i** Flow cytometry identifying the approximate trend of subset 0 (HLA-DRB1 antibody) calculated from scRNA-seq. The statistical chart in **i** displays the quantitative results. Data come from enriched NK cells (sorting with KLRF1-APC antibody) of ten participants from each of four groups. **j** qPCR analysis regarding *HLA-DRB1* levels. Data come from enriched NK cells of multiple participants in the HD (*n* = 10), EA (*n* = 13), LD (*n* = 11) and RM (*n* = 11) groups. Different letters indicate a significant decrease (*P* < 0.05), while double letters indicate no statistically significant difference between the given group and the compared groups. In **h**–**j** one-way ANOVA and Tukey’s post-hoc multiple comparisons were used. **k**–**o** Functional enrichment of the top genes in subsets 0 (**k**), 1 (**l**), 2 (**m**), 3 (**n**) and 5 (**o**). The enriched GO terms are labeled with their names. Circle size indicates the number of enriched genes, and color depth indicates the *q* value. The pivotal terms are marked in a red box. **p** Relative intergroup comparisons of GSVA scores in multiple functions between subsets 0–5. **q** Relative intergroup comparisons of GSVA scores in the endogenous antigen presentation functions from subset 0. Rows were normalized, and the transition of purple–black–yellow indicates an increase in the scores.

NK cells play a crucial role in innate defense by directly killing pathogens. We accordingly investigated the significance of NK cell taxonomy to AS lesions. After reclustering, we obtained ten NK subsets, with nine *CD56*/*NCAM1*^dim^ subsets and one *CD56*^bright^ subset (Fig. 3a–c and Supplementary Fig. 4a). We discovered that the *CD56*^bright^ cell proportion (subset 5) in HDs was the highest (Fig. 3d,e). Consistently, the proportion of *CD56*^dim^ NK cells to total PBMCs was the lowest in HDs (Supplementary Fig. 4b). After enriching NK cells, flow cytometry showed that CD56^bright^ cells were more abundant in HDs than in AS groups (Supplementary Fig. 4c,e), and among total PBMCs, KLRF1^+^CD56^dim^ cells from HDs were less abundant than those of other groups (Supplementary Fig. 4d,f); this verified the sequencing data. Compared with *CD56*^dim^ cells, *CD56*^bright^ cells possessed higher scores in negative regulation of lymphocyte-mediated immunity/NK cell activation (Supplementary Fig. 4g), and negative regulation of leukocyte-mediated immunity/leukocyte-mediated cytotoxicity/cell killing was significantly enriched in the top genes of *CD56*^bright^ subset, including *XCL1* and *CAPG* (Fig. 3f,o). This confirms noncytotoxic NK characteristics of *CD56*^bright^ cells^34^ and reveals the involvement of noncytotoxic NK cells in AS improvement.

Next, we delved into the major *CD56*^dim^ subsets. Compared with HDs, the proportion of subset 0 in EAs slightly decreased; compared with EAs, it sharply increased in LDs and decreased in RMs (Fig. 3d,e), implying that subset 0 plays a role in influencing AS severity. MHC-II complex assembly, exogenous antigen presentation and related functions were significantly enriched in the top genes of subset 0, mainly including *HLA-DRB1* (Fig. 3f,k), and exogenous antigen presentation and related functions in subset 0 were the strongest (Fig. 3p), indicating that subset 0 is NK cells participating in exogenous antigen presentation. This provides an effective supplement to previous research elucidating the APC properties of NK cells^35^. *HLA-DRB1* performs a key function in MHC-II complex assembly^36^; and its expression in NK cells decreased at the onset and was associated with AS severity (Fig. 3d,e), which was identified by flow cytometry and *HLA-DRB1* mRNA detection for enriched NK cells (Fig. 3h–j). We accordingly believe that APC-NK may be responsible for AS aggravation; this was supported by GSVA showing a trend similar to subset-0 abundances in the exogenous antigen presentation scores from HDs to LDs/RMs (Fig. 3q). In addition, the proportions of subset 1 and subset 2 sharply increased in AS groups, but both were inversely correlated with disease severity (Fig. 3d,e), implying that subsets 1 and 2 participate in AS pathogenesis but exert a protective effect on the conditions. Podosome assembly and related functions were significantly enriched in the top genes of the two subsets, including *MSN* and *LCP1* (Fig. 3f,l,m). Furthermore, the top genes of subset 2 were also associated with positive regulation of cell killing/NK-mediated cytotoxicity, including *KLRC2* and *KLRC4* (Fig. 3f,m). GSVA corresponded with the above results, describing that subsets 1 and 2 were the strongest in ADCC and positive regulation of podosome assembly (Fig. 3p). The abnormal binding of HLA-B27 to killer inhibitory receptors was implicated in AS pathogenesis^37,38^, and podosome is a structural foundation for cytoskeleton organization, causing cell–cell/matrix adhesion and determining NK cells–target cells/matrix interaction^39^. Cytotoxic NK properties of subsets 1 and 2 were accordingly supported. As the representative genes of subsets 1 and 2, *MSN* and *KLRC2* were responsible for podosome assembly and NK-mediated cytotoxicity, respectively^40,41^, and their expression was significantly higher in AS groups and varied inversely with the severity (Fig. 3g). ADCC and positive regulation of podosome assembly for subsets 1 and 2 were stronger in AS groups (Supplementary Fig. 4h). IFN-γ response and neutrophil chemotaxis/migration were significantly enriched in the top genes of subset 3, including *CCL4* and *FCER1G* (Fig. 3f,n), indicating that NK subset‑3 contributes to the neutrophil-dependent first line of defense. This is consistent with previous reports showing that NK cells promote neutrophil activation, survival and recruitment.^42,43^. NK subset 3 also exhibited the strongest neutrophil chemotaxis/migration (Fig. 3p). Subset-3 proportions were lower in AS groups than in HDs (Fig. 3d,e), implying that decreased subset-3 abundance contributes to AS pathogenesis. As a marker, *FCER1G* is responsible for the bacterial defense response and neutrophil-mediated innate immunity^41^; and its expression in AS groups was lower than that in HDs (Fig. 3f,g). We therefore inferred that impaired initial defense in patients with AS may be advantageous for bacterial infection and trigger subsequent immune responses, further confirming the rationality of sulfasalazine in AS treatment. Both subset-4 abundances and the top genes (*CD3D*/*E*/*G*) showed a sequential decrease from HDs to LDs/RMs (Fig. 3d–g), suggesting that accelerated NK cell development occurs during AS onset and progression, with *CD3D*/*E*/*G* serving as markers of pre‑NK cells^44^.

### Antigen presentation characteristics across AS conditions

**Fig. 4:**
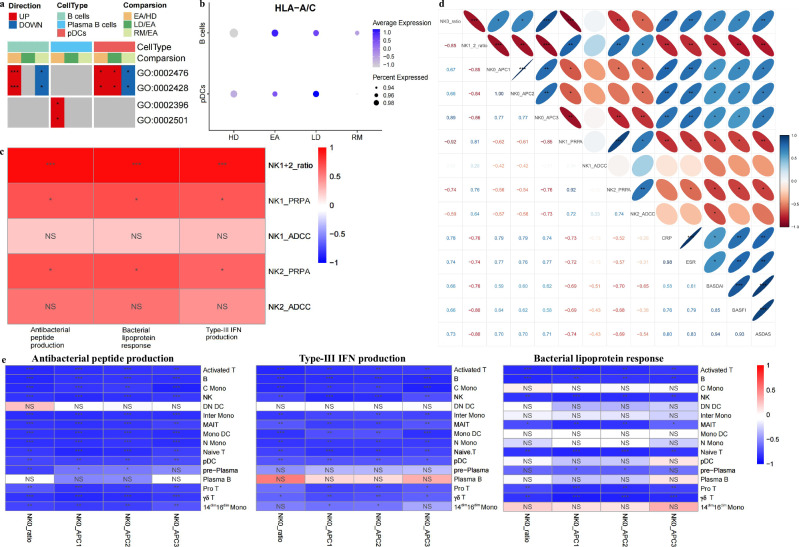
Antigen presentation characteristics across AS conditions. **a** Functional enrichment showing several antigen presentation functions for B cells, plasma B cells and pDCs between corresponding groups. Red indicates the functions associated with upregulated genes, and blue indicates the opposite. The number of asterisks indicates the *q* value. **b** The expression distribution of HLA-A/C in B cells and pDCs across four groups. **c** Correlation analysis between the proportions of NK subsets‑1/2, their positive regulation of podosome assembly (PRPA) and ADCC scores, and the innate defense function scores of NK cells from patients. **d** Correlation analysis between NK-subset-0 cell proportions, NK-subsets-1/2 cell proportions, NK-subset-0-mediated exogenous antigen presentation (APC1–3) scores, NK-subset-1/2-mediated PRPA and ADCC scores and various clinical parameters from patients. **e** Correlation analysis between NK-subset-0 cell proportions, NK-subset-0-mediated APC1–3 scores and innate defense function scores of 16 main cell types from patients. In **b**–**e** the number of asterisks indicates the *q* value, and different colors indicate the *R* value. NS, not significant.

There seems to be a link between innate defense and antigen presentation. We therefore provided a comprehensive characterization of endogenous and exogenous antigen presentation across different AS conditions. GO analysis showed that, compared with HDs, EAs exhibited significantly upregulated functions in MHC-IB-dependent endogenous antigen presentation for B cells and pDCs, and MHC protein complex assembly for plasma B cells (Fig. 4a). The total expression of responsible genes, HLA-A/C, increased in EAs (Fig. 4b). However, based on EAs, all parameters in RMs decreased (Fig. 4a,b). B cells and pDCs may serve as the valuable endogenous APCs causing AS onset; these observations advance previous research demonstrating epitope peptide presentation from HLA-B8-expressing B cells to cytotoxic T cells and the relationship between DCs and AS^45,46^.

We documented that overall innate defense scores and cytotoxic NK subset abundance shared similar trends and they varied inversely with APC-NK abundance and their presentation functions. Subsets 1 and 2 showed the highest scores in innate defense functions (Fig. 3p), and their functions were stronger in AS groups (Supplementary Fig. 4h). The involvement of NK-dependent cytotoxicity in innate defense is clarified, conforming to conventional understandings^47–49^. As shown in Fig. 4c, the total proportions of subsets 1 and 2 and their podosome assembly functions were positively correlated with NK-related innate defense scores, indicating that NK-dependent cytotoxicity contributes to AS onset but also to condition improvement. Furthermore, AS lesions can produce numerous proinflammatory and dead cells, suggesting that NK-mediated cytotoxicity helps establish postimmune homeostasis by scavenging redundant cells, conforming to the results showing that apoptotic signaling for most types in EAs was weaker than that in LDs but stronger than that in RMs (Fig. 2a and Supplementary Fig. 2a). As shown in Fig. 4d, the proportions of subset 0 and their antigen presentation functions were negatively correlated with the abundance of subsets 1 and 2, positive regulation of podosome assembly scores for subsets 1 and 2, and ADCC scores for subset 2, confirming the trade-off relationship between APC-like NK cells and cytotoxic NK cells. The conflict between two NK cell types suggests a connection between exogenous antigen presentation and innate defense. As expected, the scores for three innate defense functions in most cell types were negatively correlated with subset-0 proportions and their presentation scores (Fig. 4e). Remarkably, subset-0 abundance and their presentation scores were positively correlated with various clinical parameters, and cytotoxic NK abundance and their podosome assembly functions were negatively correlated with ESR, BASDAI, BASFI and Ankylosing Spondylitis Disease Activity Scores (ASDAS) (Fig. 4d). The significance of NK-dependent exogenous antigen presentation for innate defense and AS severity was described.

### The impact of APC-NK on CD4^+^ T cell parameters

**Fig. 5:**
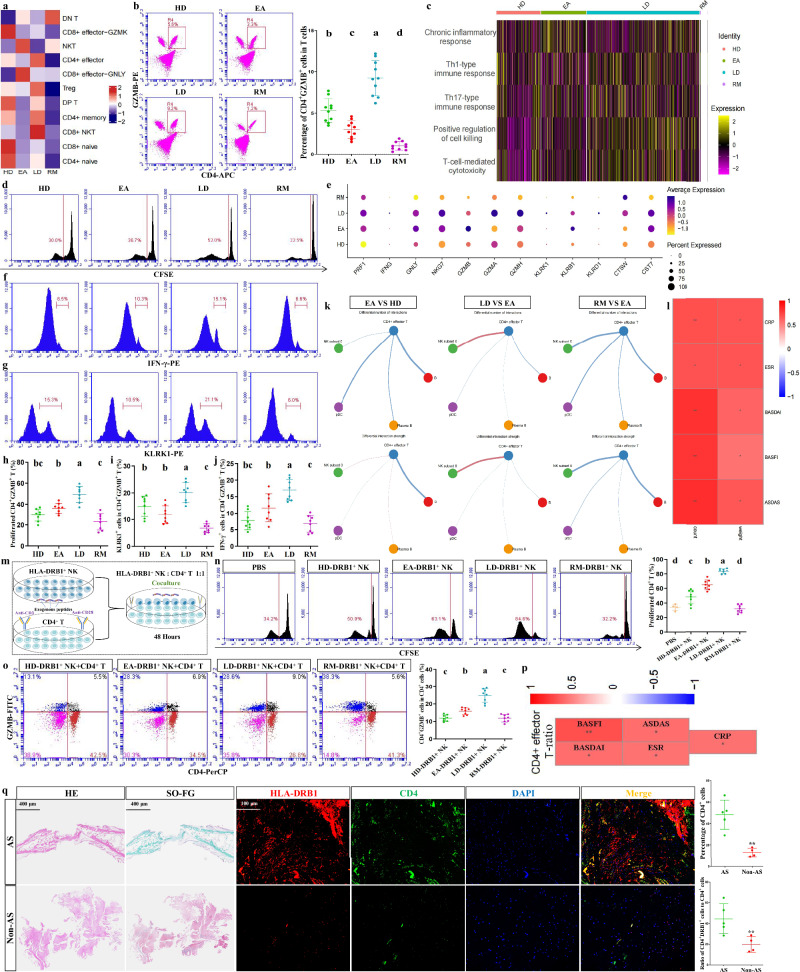
The impact of APC-NK on CD4^+^ T cell parameters. **a** Intergroup preferences of various T cell subtypes. Columns were normalized, and the transition of blue–white–red indicates an increase in the proportion. **b** Flow cytometry identifying the approximate trend of CD4^+^ effector T cells (CD4 and GZMB antibodies) calculated from scRNA-seq. Data come from enriched T cells (sorting with CD3-FITC antibody) of 10 participants in each of four groups. Different letters indicate a significant decrease (*P* < 0.05). One-way ANOVA and Tukey’s post-hoc multiple comparisons. **c** Relative intergroup comparisons of GSVA scores in multiple functions from CD4^+^ effector T cells. Rows were normalized, and the transition of purple–black–yellow indicates an increase in the scores. **d** CFSE assays showing the proliferative potential of enriched CD4^+^ T cells ^(^sorting with CD3-PE and CD4-APC antibodies) from four groups (*n* = 8) in the presence of CD3 and CD28 antibodies. **e** The expression distribution of cytotoxic genes in CD4^+^ effector T cells across four groups. **f**,**g** Flow cytometry showing the proportions of positive cells for two cytotoxic molecules (IFN-γ (**f**) and KLRK1 (**g**)) in CD4^+^GZMB^+^ T cells (sorting with CD3-FITC, CD4-APC and GZMB-PerCP antibodies) across four groups (*n* = 8). **h**–**j** Quantitative results for **d** (**h**), **f** (**i**) and **g** (**j**). **k** Intercellular communications showing differential action number and strength from B cells, plasma B cells, pDCs and NK subset 0 to CD4^+^ effector T cells between corresponding groups. **l** Correlation analysis between the number (count) and strength (weight) of signal transmission from APC-NK to CD4^+^ effector T cells and various clinical parameters from patients. The number of asterisks indicates the *q* value, and different colors indicate the *R* value. **m** Coculture model simulating the presentation of exogenous peptides by HLA-DRB1^+^ NK (sorting with KLRF1-APC and HLA-DRB1-PE antibodies) to CD4^+^ T cells. **n** CFSE assays showing the proliferative ability of CD4^+^ T cells cocultured with HLA-DRB1^+^ NK cells from four groups (*n* = 8) or PBS (*n* = 4). **o** Flow cytometry showing the proportions of CD4^+^GZMB^+^ cells in the cocultured cells for each group (*n* = 8). Quantitative results were based on the ratio of CD4^+^GZMB^+^ cells to CD4^+^ cells. **p** Correlation analysis between CD4^+^ effector T cell proportions and various clinical parameters from patients. The number of asterisks indicates the *q* value, and different colors indicate the *R* value. **q** H&E, SO-FG and HLA-DRB1/CD4 co-immunostaining (including quantification of HLA-DRB1 and CD4 fluorescence expression) in the ligament tissues from patients with AS and non-AS patients. Scale bars, 400 µm or 100 µm. Data in **q** come from the collected ligament samples from non-AS patients (*n* = 4) and patients with AS (*n* = 5) during surgeries. Different letters indicate a significant decrease (*P* < 0.05), while double letters indicate no statistically significant difference between the given group and the compared groups. In **b**, **h**–**j**, **n** and **o** one-way ANOVA and Tukey’s post-hoc multiple comparisons were used. In **q** **P < 0.01 by Student’s *t*-tests.

The impact of APC-like NK cells and their antigen-presentation functions on AS disease-state alterations was elucidated. The object of exogenous antigen presentation is CD4^+^ T cells. We accordingly conducted T cell reclustering analysis. After reclustering, we obtained 11 subtypes according to the distribution of classical markers (Supplementary Fig. 5a–d). We discovered that five effector T cell subtypes (CD4^+^ effector, CD8^+^ effector-GNLY, CD8^+^ effector-GZMK, CD8^+^ NKT and NKT cells) were specifically involved in AS onset and deterioration (Supplementary Fig. 5e,f), suggesting that AS treatment at different stages should take into account T cell specificity. However, AS remission is characterized by the overall reduction in effector T cell abundance and their functions, supporting the crucial role of stable T cell immunity in AS remission (Supplementary Figs. 5e,f and 6a,b). Among CD4^+^ or CD8^+^ cells, the trend of naive cells demonstrated that AS is characterized by rapid T cell maturation; this was validated by *CCR7* mRNA detection in enriched CD4^+^ or CD8^+^ T cells (Supplementary Fig. 5g–j). CD4^+^ effector-subtype proportions were associated with AS severity (Fig. 5a); this was validated by flow cytometry showing the trend of CD4^+^GZMB^+^ cells in enriched T cells (Fig. 5b). GSVA showed that positive regulation of cell killing and T-cell-mediated cytotoxicity for CD4^+^ effector T cells strengthened in EAs and were associated with severity (Fig. 5c and Supplementary Fig. 6b). These findings suggest that CD4^+^ effector T cells may represent a potential therapeutic target.

Notably, CD4^+^ effector T cell abundance and APC-NK shared a consistent trend; this implies a connection between the two. CFSE assays showed that CD4^+^ T cell proliferation from LDs increased and that of RMs decreased (Fig. 5d,h), indicating that CD4^+^ T cell proliferative potential corresponds to AS conditions; this validates CD4^+^ effector T cell abundance associated with AS severity. The expression trends of various cytotoxic genes in CD4^+^ effector T cells were similar to their cytotoxic functions (Fig. 5e); this was similar to flow cytometry showing that IFN-γ^+^ or KLRK1^+^ cell abundance for CD4^+^GZMB^+^ T clusters increased in LDs and decreased in RMs (Fig. 5f,g,i,j). The involvement of CD4^+^ T-dependent cytotoxicity in AS severity was therefore demonstrated. In addition, GSVA showed that among, three representative cytokines for AS lesions^5^^,^^6^^,^^50^, IL-17 or IFN-γ production in CD4^+^ effector T cells was stronger in AS groups than in HDs, and the production of all three cytokines was associated with disease severity (Supplementary Fig. 7a), suggesting that, besides cytotoxicity, activated CD4^+^ T cells serve as the producers for key cytokines that alter AS conditions, and further supporting their targetability. Based on EAs, CD4^+^ effector T cells showed an increase in signal input from APC-NK in LDs while showing a reduced input in RMs (Fig. 5k), indicating that signal transmission from APC-NK to CD4^+^ effector T cells is a crucial factor in AS severity; this was also validated by the observation showing that signal transmission number and strength from APC-NK to CD4^+^ effector T cells were positively correlated with various clinical parameters (Fig. 5l).

Flow cytometry showed that HLA-DPB1^+^ cells were more abundant in enriched HLA-DRB1^+^ NK cells than in HLA-DRB1^−^ NK cells (Supplementary Fig. 7b), confirming the results in Fig. 3f and supporting the reliability of HLA-DRB1^+^ NK clusters. To validate the impact of APC-NK on CD4^+^ T cells, we established coculture systems for HLA-DRB1^+^ NK cells and enriched CD4^+^ T cells (Fig. 5m). CFSE assays revealed that coculture with HLA-DRB1^+^ NK cells indeed enhanced CD4^+^ T cell proliferation (Fig. 5n). CD4^+^ T cells cocultured with HLA-DRB1^+^ NK cells from EAs showed stronger proliferation than those for HDs, and HLA-DRB1^+^ NK-contributed cell proliferation was associated with the severity (Fig. 5n), supporting that the effects of APC-NK on CD4^+^ T cell abundance can affect AS conditions. Similarly, GZMB^+^ cell abundance among CD4^+^ T clusters was associated with severity (Fig. 5o and Supplementary Fig. 7c). Intercellular communication results were accordingly supported. Clinical parameters were positively correlated with CD4^+^ effector T cell abundance (Fig. 5p), further revealing their contribution to AS severity.

Next, we further evaluated the contribution of APC-NK–CD4^+^ T cell interactions to AS lesions using clinical histology. As shown in Fig. 5q, AS-ligament tissues presented massive new-bone formation. Moreover, CD4 and HLA-DRB1 fluorescence expression in AS-ligament tissues (HLA-DRB1 is displayed as HLA-DRB1/CD4 overlapping fluorescence) was significantly stronger than that of non-AS patients **(**Fig. 5q), further supporting the role of APC-NK–CD4^+^ T cell interactions in AS lesions from a histological perspective. In addition, the association between CD4^+^ T cell expansion and AS lesions was also histologically confirmed.

### The significance of APC-NK–CD4^+^ T communication for AS-like phenotypes

**Fig. 6:**
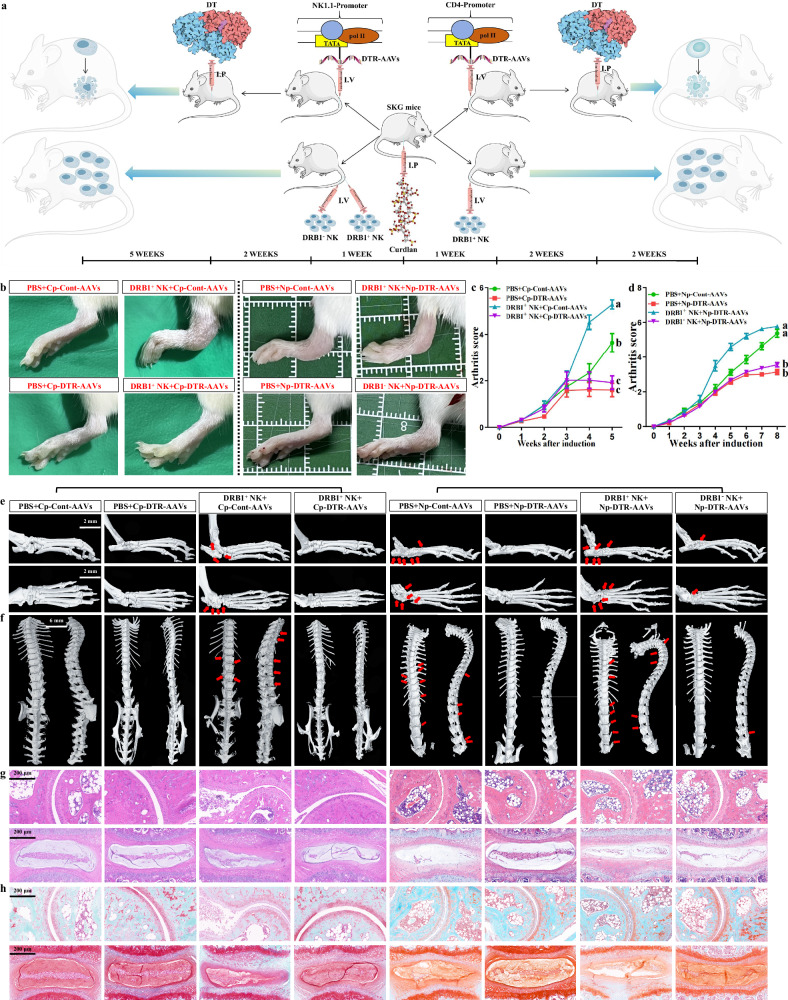
The significance of APC-NK–CD4^+^ T communication for AS-like phenotypes. **a** The administration model of NK1.1-promoter-*DTR*-AAVs, CD4-promoter-*DTR*-AAVs and different clusters of human NK cells. After curdlan induction, the above AAVs and enriched HLA-DRB1^+^ or HLA-DRB1^−^ NK cells were applied to treat AS modeling mice by tail-vein injection, and diphtheria toxin was injected intraperitoneally 2 weeks after AAV administration to promote target-cell death. **b** Comparison of ankle-swelling degree between four groups of mice on the left and four groups of mice on the right (*n* = 5). **c**,**d** The mice from each group were evaluated with clinical arthritis scores once a week, and the overall trend was compared between corresponding groups (**c** CD4-promoter-*DTR*-AAVs-related groups; **d** NK1.1-promoter-*DTR*-AAVs-related groups) (*n* = 5). The different letters indicate a significant decrease with *P* < 0.05. In **c**, two-way ANOVA and Tukey’s post-hoc multiple comparisons were used. In **d** ***P < 0.001 by Student’s *t*-tests. **e**,**f** Micro-CT showing the formation of osteophytes in the ankle joints (**e**) and spines (**f**) (indicated by red arrows) as well as joint-degeneration degree across each group (*n* = 5). Scale bars, 2 mm (ankle joint) or 6 mm (spine). **g**,**h** H&E (**g**) or SO-FG (**h**) staining showing ankle-joint destruction, spinal degeneration and tissue ossification (displayed as green area) across each group (*n* = 5). The experiment was independently repeated in each group of mice. Scale bar, 200 µm. DT, diphtheria toxin; Cp-Cont-AAVs, CD4-promoter-Cont-AAVs; Cp-DTR-AAVs, CD4-promoter-*DTR*-AAVs; Np-Cont-AAVs, NK1.1-promoter-Cont-AAVs; Np-DTR-AAVs, NK1.1-promoter-*DTR*-AAVs; I.P, intraperitoneal injection; I.V, intravenous (tail-vein) injection.

Subsequently, we applied SKG mice with curdlan induction, HLA-DRB1^+^ NK cells and corresponding AAVs that induce specific cell death to observe the impact of NK-dependent exogenous antigen presentation on AS-like phenotypes (Fig. 6a). NK cell exhaustion or CD4^+^ T cell exhaustion in vivo caused by NK1.1-promoter- or CD4-promoter-diphtheria toxin receptor (*DTR*)-AAVs was verified, showing that both treatments respectively reduced NK1.1^+^ cell and CD4^+^ cell abundance in PBMCs and promoted the apoptosis of enriched cells (Supplementary Fig. 8a,b). Furthermore, curdlan administration increased CD4^+^GZMB^+^ T cell abundance in vivo, and the implantation of human HLA-DRB1^+^ NK cells further increased that of curdlan-treated mice while not affecting that of curdlan-untreated mice (Supplementary Fig. 8c). By contrast, AAV-induced NK cell exhaustion decreased CD4^+^GZMB^+^ T cell abundance in vivo (Supplementary Fig. 8d). The stimulation of CD4^+^ T cell activation by APC-NK was accordingly supported in vivo. As expected, HLA-DRB1^+^ NK cell implantation did advance the modeling of AS-like mice. In addition to the pronounced stiffness observed when peeling off the spines, mice implanted with HLA-DRB1^+^ NK cells exhibited marked ankle-joint swelling by week 5, indicating successful model establishment (Fig. 6b). The arthritis scores significantly increased with the above treatment (Fig. 6c). As shown on micro-CT, mice implanted with HLA-DRB1^+^ NK cells exhibited obvious tissue degeneration and osteophyte formation in the ankles and spines (Fig. 6e,f and Supplementary Fig. 8c). Histological assessment also showed that this treatment led to premature ankle-joint ossification and destruction, as well as spinal ossification and degeneration (Fig. 6g,h). Meanwhile, these changes were inconspicuous in conventionally modeled mice (Fig. 6b,c,e–h). However, CD4^+^ T cell exhaustion abolished all changes induced by the cell transfer (Fig. 6b,c,e–h). NK cell exhaustion also improved ankle-joint swelling and decreased arthritis scores in AS modeling mice receiving sufficient induction (Fig. 6b,d). NK cell exhaustion effectively improved tissue ossification and destruction in the ankles and spines (Fig. 6e–h). Subsequently, both HLA-DRB1^+^ and HLA-DRB1^−^ NK cells were implanted into mice with NK cell exhaustion to further validate the precision effect of APC-NK on the above phenotypes. As shown in Fig. 6b,d,e–h, HLA-DRB1^+^ NK cell implantation recovered AS-like phenotypes repressed by NK cell exhaustion while HLA-DRB1^−^ NK cell implantation had no obvious effects. Given the non-APC-NK attribute of HLA-DRB1^−^ NK cells, these results suggest the specificity of APC-NK on AS lesions. No obvious graft-versus-host disease was found, such as rash, diarrhea or anorexia. Moreover, mouse body weight and blood indicators showed nonsignificant alterations with NK cell transfer (Supplementary Fig. 9). Therefore, the interference of heterologous immune rejection on animal experiments was excluded. These results supported the contribution of APC-NK–CD4^+^ T signal transmission to AS-like alterations.

### HLA-DPB1/DPA1 in APC-NK participates in AS aggravation

**Fig. 7:**
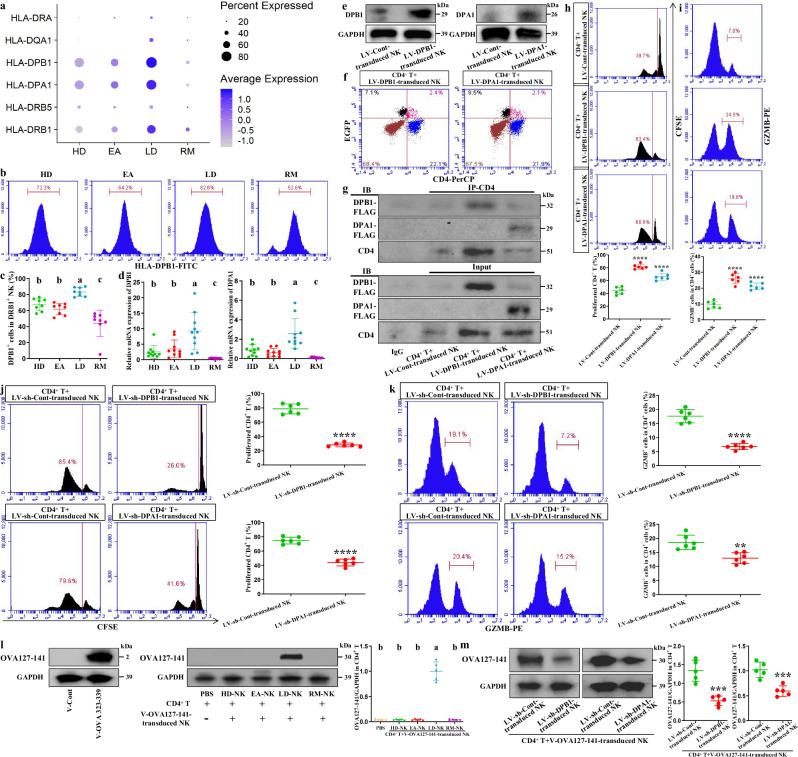
HLA-DPB1/DPA1 in APC-NK participates in AS aggravation. **a** The expression distribution of various HLA genes in NK subset 0 across four groups. **b**,**c** Flow cytometry showing the proportions of HLA-DPB1-positive cells in HLA-DRB1^+^ NK cells across four groups (*n* = 8). **d** qPCR analysis regarding *HLA-DPB1* and *HLA-DPA1* levels from enriched HLA-DRB1^+^ NK cells of 10 participants in each of four groups. **e** Western blotting validation of HLA-DPB1- or HLA-DPA1-overexpressed NK cells. **f** Flow cytometry showing the proportions of EGFP^+^CD4^+^ cells in the cocultured cells including HLA-DPB1- or HLA-DPA1-overexpressed NK cells and CD4^+^ T cells (sorting with CD3-PE and CD4-APC antibodies). Data come from three repeated experiments with similar results. **g** Co-immunoprecipitation analysis regarding the interaction of CD4 and Flag in the cocultured cells including HLA-DPB1- or HLA-DPA1-overexpressed NK cells and CD4^+^ T cells. Data come from three repeated experiments with unanimous results. IP indicates immunoprecipitating antibody, and IB indicates immunoblotting antibody. **h** CFSE assays showing the proliferative ability of CD4^+^ T cells cocultured with HLA-DPB1- or HLA-DPA1-overexpressed NK cells. **i** Flow cytometry showing the proportions of GZMB^+^ cells in the CD4^+^ T cells (sorting with CD3-FITC and CD4-APC antibodies) cocultured with HLA-DPB1- or HLA-DPA1-overexpressed NK cells. **j** CFSE assays showing the proliferative ability of CD4^+^ T cells cocultured with HLA-DPB1- or HLA-DPA1-silenced NK cells. **k** Flow cytometry showing the proportions of GZMB^+^ cells in the CD4^+^ T cells cocultured with HLA-DPB1- or HLA-DPA1-silenced NK cells. Data come from six independent samples from HDs (**h** and **i**) or LDs (**j** and **k**). **l** Western blotting showing OVA127-141 expression in CD4^+^ T cells cocultured with OVA127-141-overexpressed NK cells from four groups (*n* = 5) or PBS (*n* = 5). **m** Western blotting showing OVA127-141 expression in CD4^+^ T cells cocultured with OVA127-141-overexpressed plus HLA-DPB1- or HLA-DPA1-silenced NK cells. Data come from five independent samples from LDs. **P < 0.01; ***P < 0.001; ****P < 0.0001 by Student’s *t*-tests. The different letters indicate a significant decrease with *P* < 0.05. In **c**, **d** and **l** one-way ANOVA and Tukey’s post-hoc multiple comparisons were used. LV, lentivirus; V, vector.

Finally, we attempted to identify meaningful exogenous antigen-presenting molecules in APC-NK. Through intercellular communications, we discovered that HLA-DPB1/DPA1 in APC-NK from LDs had maximum communication strength with CD4 in CD4^+^ effector T cells (Supplementary Fig. 10a). As a T cell coreceptor, CD4 synergizes with the T cell receptor to convert antigen signals presented by MHC class II molecules into effective T cell activation, suggesting the value of the above molecules. As expected, their levels in APC-NK were similar to APC-NK abundance and their presentation scores in trend (Fig. 7a); this was supported by the association between HLA-DPB1^+^ cell abundance/*HLA-DPB1*/*DPA1* mRNA levels and severity (Fig. 7b–d), thereby validating communication results. To investigate the impact of HLA-DPB1/DPA1 on CD4^+^ T cells, we used the lentiviral technique to overexpress these genes in NK cells from HDs and further established coculture systems with CD4^+^ T cells. The overexpression efficiency of two genes is shown in Fig. 7e. Flow cytometry demonstrated that HLA-DPB1/DPA1 serve as the key HLA molecules linking NK cells with CD4^+^ T cells by showing that lentivirus-bound EGFP and CD4 double-positive cells existed in the coculture environment (Fig. 7f). In addition, HLA-DRB1^+^ NK-CD4^+^ T mixed cells from LDs showed CD4-DPB1/DPA1 interaction that was absent in single CD4^+^ T cell lysate (Supplementary Fig. 10b); this was supported by the interaction between lentiviruses-binding Flag from infected cells and CD4 (Fig. 7g). Among four conditions, CD4-DPB1/DPA1 interaction for mixed cells exclusively appeared in LDs (Supplementary Fig. 10c), corresponding to communication results in Supplementary Fig. 10a. Moreover, HLA-DPB1/DPA1-overexpressed NK cells both increased the proliferation and GZMB^+^ cell abundance in cocultured CD4^+^ T cells, and HLA-DPB1/DPA1-silenced NK cells both inhibited the above parameters from LDs (Fig. 7h–k and Supplementary Fig. 10d), supporting the significance of HLA-DPB1/DPA1 for CD4^+^ T cell activation and AS severity. Here, we applied ovalbumin peptides to initiate antigen presentation from APC-NK to CD4^+^ T cells. Therefore, we need to clarify the exogenous antigens presented by APC-NK via HLA-DPB1/DPA1. Relying on the MHC-II Binding Prediction website, we discovered that OVA127-141 peptide exhibits strong binding affinity across multiple alleles of DPB1/DPA1 (Supplementary Table 5**:** %Rank<2% indicates strong MHC binder). We continued to confirm the association between APC-NK-DPB1/DPA1 and OVA127-141 using the gene transduction technique. As shown in Fig. 7l, OVA127-141-overexpressed NK cells from LDs effectively increased the expression of OVA127-141 in cocultured CD4^+^ T cells while those from other groups had no significant effect. Furthermore, HLA-DPB1/DPA1 silencing in LD NK cells both inhibited OVA127-141 expression in cocultured CD4^+^ T cells (Fig. 7m). The role of the OVA127-141-HLA-DPB1/DPA1 complex in APC-NK-dependent exogenous antigen presentation targeting CD4^+^ T cells was supported. We accordingly infer that HLA-DPB1/DPA1 contributes to AS aggravation by mediating APC-NK–CD4^+^ T cell antigen presentation.

## Discussion

Here, we obtained an integral AS-associated immune-response profile at single-cell resolution, illustrating the dynamic features of cellular responses across different conditions. Generally, AS onset was defined as effective immune activation. Compared with the onset, AS aggravation presented marked complexity, including various upregulation and downregulation. AS remission was also characterized by the coexistence of functional enhancements and impairments. Therefore, the global immune response is not synchronized with AS development; this reveals how AS differs from other autoimmune diseases, suggesting that AS deterioration cannot be sattributed solely to immune activation. Conventional immunosuppressants, including glucocorticoids, methotrexate and leflunomide, are not recommended for routine use during AS course^5,31^; this distinguishes AS from other rheumatic diseases. The uniqueness of AS treatment can be explained by our scRNA-seq data.

**Fig. 8:**
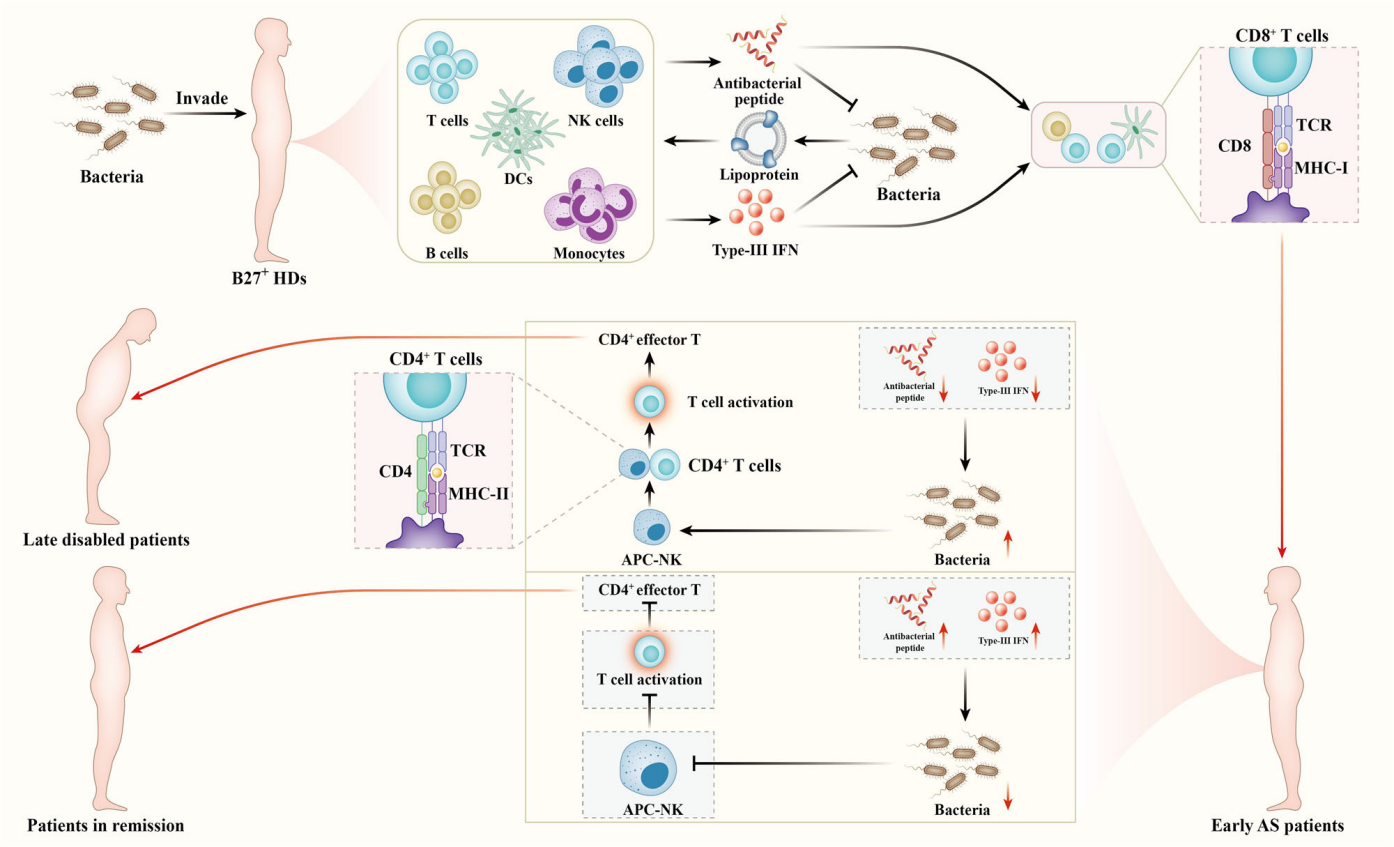
The working model for our study. With bacterial infection, the immunocytes in HLA-B27+ HDs possess response to bacterial lipoprotein, and produce antibacterial peptides and type-III IFNs; this promotes APCs to drive endogenous antigen presentation and cause CD8+ T-cell activation, resulting in AS onset. The reduction in antibacterial peptide and type-III IFN production enhances bacterial infection; this promotes APC-NK to drive exogenous antigen presentation and cause CD4+ T-cell activation, further resulting in AS aggravation. However, the increase in antibacterial peptide and type-III IFN production weakens bacterial infection; this inhibits APC-NK-drived exogenous antigen presentation and CD4+ T-cell activation, resulting in AS remission.

Based on information from scRNA-seq, we identified a novel and intriguing scientific pattern across AS conditions. First, we discovered that innate antibacterial defense strengthens with the onset but is favorable for AS-condition improvement in general. It is well known that AS is related to various pathogenic infections^27,28,51^, and the effects of infection on AS lesions involve many hypotheses, most of which are primarily based on HLA-B27^7–9^. However, the impact of infection on AS pathological changes remains poorly understood, and addressing it could benefit from the concept of 'infection-driven immune responses' across AS conditions. Furthermore, previous studies focused only on the role of infection-induced immunity in AS occurrence from the perspective of sensitizing T lymphocytes to identify arthritogenic peptides but overlooked its effects on subsequent development^32,52,53^. Our research elucidates the relationship between innate defense and outcomes, suggesting the protective effects of antibacterial ability on patients. Given that antibacterial activity determines pathogenic bacterial survival and exogenous antigen levels, APC-NK parameters opposing innate defense, along with changes in CD4^+^ effector T cell parameters, revealed a general principle: different degrees of antibacterial activity influence the contribution of NK-dependent exogenous antigen presentation to AS severity, with enhanced activity promoting AS aggravation and reduced activity producing the opposite effect. Then, APC-NK make CD4^+^ T cell activation influence different outcomes by antigen presentation to CD4^+^ T cells. Currently, the role of CD4^+^ T cells in AS pathogenesis is mainly elucidated in terms of T helper cell response and T regulatory functions^54–57^. However, the relationship between antigen presentation received by CD4^+^ T cells and AS pathogenesis has not been clearly demonstrated. Here, the association between the two was indicated, and the biological patterns of antigen presentation targeting CD4^+^ T cells involved in AS outcomes were clarified. The antigen-presenting functions of HLA-DR-expressing NK cells were supported by several studies^35^. Our study not only revealed an APC‑NK cluster primarily expressing HLA‑DRB1, but also confirmed its crucial contribution to AS severity. In vivo assays further provided key evidence supporting this principle, demonstrating the significance of APC-NK–CD4^+^ T signal transmission to AS-like phenotypes. Notably, curdlan used to induce AS modeling mice is an aggregate containing β-glucan, which is a major component of bacterial and fungal cell walls^17^. Previous research simply linked CD4^+^ T-cell-mediated autoimmunity and curdlan’s pathogenicity but ignored the intrinsic mechanism underlying their association^17^. Our data present a crucial biological explanation for curdlan-triggered AS-like phenotypes from an antigen-presenting perspective and effectively links curdlan’s pathogenicity with activated CD4^+^ T cells, thus confirming at the animal level the role of APC-NK-dependent antigen presentation in AS aggravation. Due to technological limitations, AAVs targeting AS modeling mice resulted in overall depletion of NK cells and failed to highlight the effect of APC-NK, which is a limitation in in vivo assays. However, the in vivo effects caused by NK cell exhaustion confirm our conclusion, which supports the feasibility of using the AAVs. Importantly, we discovered that human APC-NK serves as an effective compensation for NK cell exhaustion whereas non-APC-NK is ineffective; this effectively addresses the shortcomings of the AAVs. Moreover, two HLA genes, HLA-DPA1/DPB1, were supported to play a significant role in APC-NK–CD4^+^ T cell antigen presentation, and OVA127-141 can be presented by APC-NK relying on HLA-DPA1/DPB1, providing potential targets for improving conditions. We accordingly infer that repressing HLA-DPA1/DPB1 expression in NK cells may be a candidate strategy for preventing disability and OVA127-141 has significant implications for AS severity; this deserves future exploration. Moreover, several known APCs exhibited strong endogenous antigen presentation during disease onset, corresponding to enhanced antibacterial peptide and type-III IFN production in early-stage patients; this again highlights the significance of abnormal antigen processing for AS onset^9,32,33^. However, all APCs here demonstrated clinical remission linked with weak antigen presentation, indicating the significance of clearing self antigens and cytotoxic NK cells for condition improvement. The cytotoxic NK clusters identified here warrant further investigation. Together, CD8^+^ T cell or CD4^+^ T cell activation was responsible for AS occurrence or progression, respectively; this reminds us that AS treatment can achieve better efficacy based on T cell specificity at different stages. Therefore, our comprehensive analysis of peripheral immunocytes provides a clear overview of innate antibacterial defense, exogenous antigen presentation and CD4^+^ T cell activation signaling involved in AS outcomes. The main working model is presented in Fig. 8. Moreover, our comprehensive analysis revealed that infection acts as a risk factor for both disease onset and aggravation, despite differences in specific mechanisms, while infection improvement contributes to remission; this was supported by MR analysis. The impairment of NK-related initial defense is conducive to AS lesions, further supporting the above theory. Accordingly, the therapeutic value of sulfasalazine in AS treatment should continue to be recognized.

Based on multicellular descriptions, we presented a comprehensive view of immunological profiles across AS conditions and provided important insights into the specifics for AS onset, aggravation and remission. Importantly, AS-associated innate defense and antigen presentation patterns were clarified. APC-NK is responsible for AS-condition alterations by bridging innate defense and CD4^+^ T cell activation, revealing that APC-NK deletion is a promising strategy for preventing ankylosing deformities; this sheds lights on translational research. Medication guidelines regarding sulfasalazine also received insights from scRNA-seq data. Here, our immunomics data provide an important reference for understanding AS-associated immune responses in depth by demonstrating that the unconventional functions of classic cytotoxic cells contribute to CD4^+^ T cell activation and AS severity.

## Supplementary information

Supplementary Information

## Data Availability

The scRNA-seq data are publicly available via the NCBI Sequence-Read-Archive (SRA) using the accession number PRJNA1168183. Other data supporting the findings in this study are available upon reasonable request.
