# Supplementary material for: Single-immunocyte transcriptomics reveal the role of natural killer cell-dependent exogenous antigen presentation in ankylosing spondylitis severity
Source: Exp Mol Med. 2026 Jan 28;58(1):254–71. doi: 10.1038/s12276-025-01619-6 (PMC12868835; doi:10.1038/s12276-025-01619-6)
Supplement: Supplementary file 1 — Supplementary Information [file 12276_2025_1619_MOESM1_ESM.pdf]

Supplementary Figures

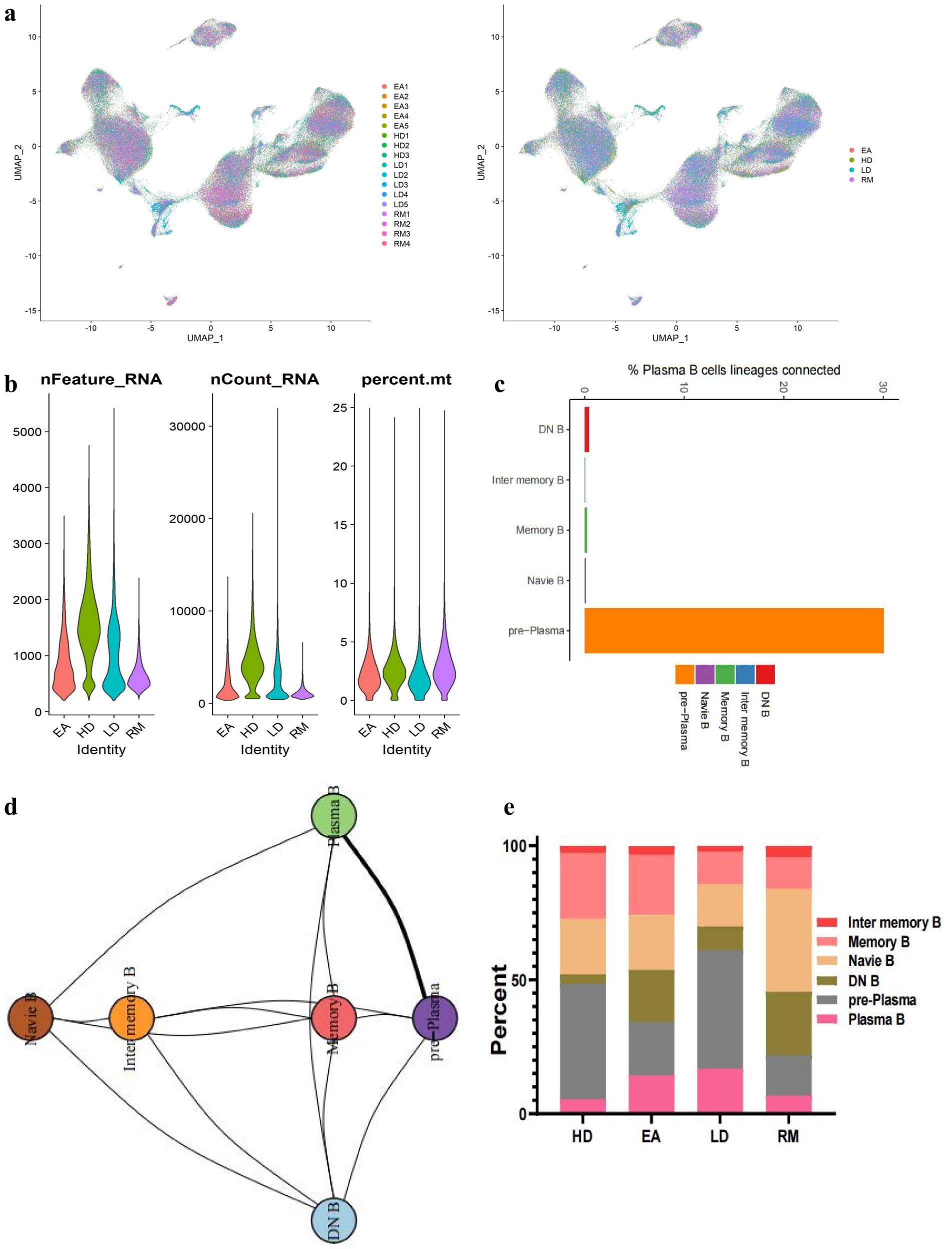

Supplementary Fig. 1 Clustering quality of PBMCs for HLA-B27$^+$ healthy donors and AS patients. a UMAP projection of all cells colored at sample levels (left) and group levels (right). Approximately 3.7 billion unique transcripts were obtained from 118,334 PBMCs from all samples, among which 20747 cells (17.5%) were from HDs, 33434 cells (28.3%) were from EAs, 36680 cells (31.0%) were from LDs, and 27473 cells (23.2%) were from RMs. **b** Violin chart showing gene counts (left), UMIs (middle) and percentage of mitochondrial genes (right) in all cells. The above parameters in each group are within an acceptable range, indicating the effectiveness and integrability of scRNA-seq data. **c** The overall percentage of IgG-clonal lineages in plasma B cells shared with pre-plasma, naive B, memory B, intermemory B and DN B cells. **d** Clonal connectivity between various types of B cells. **e** Respective proportions of B-cell subtypes and two effector B cells to total B cells across four groups.

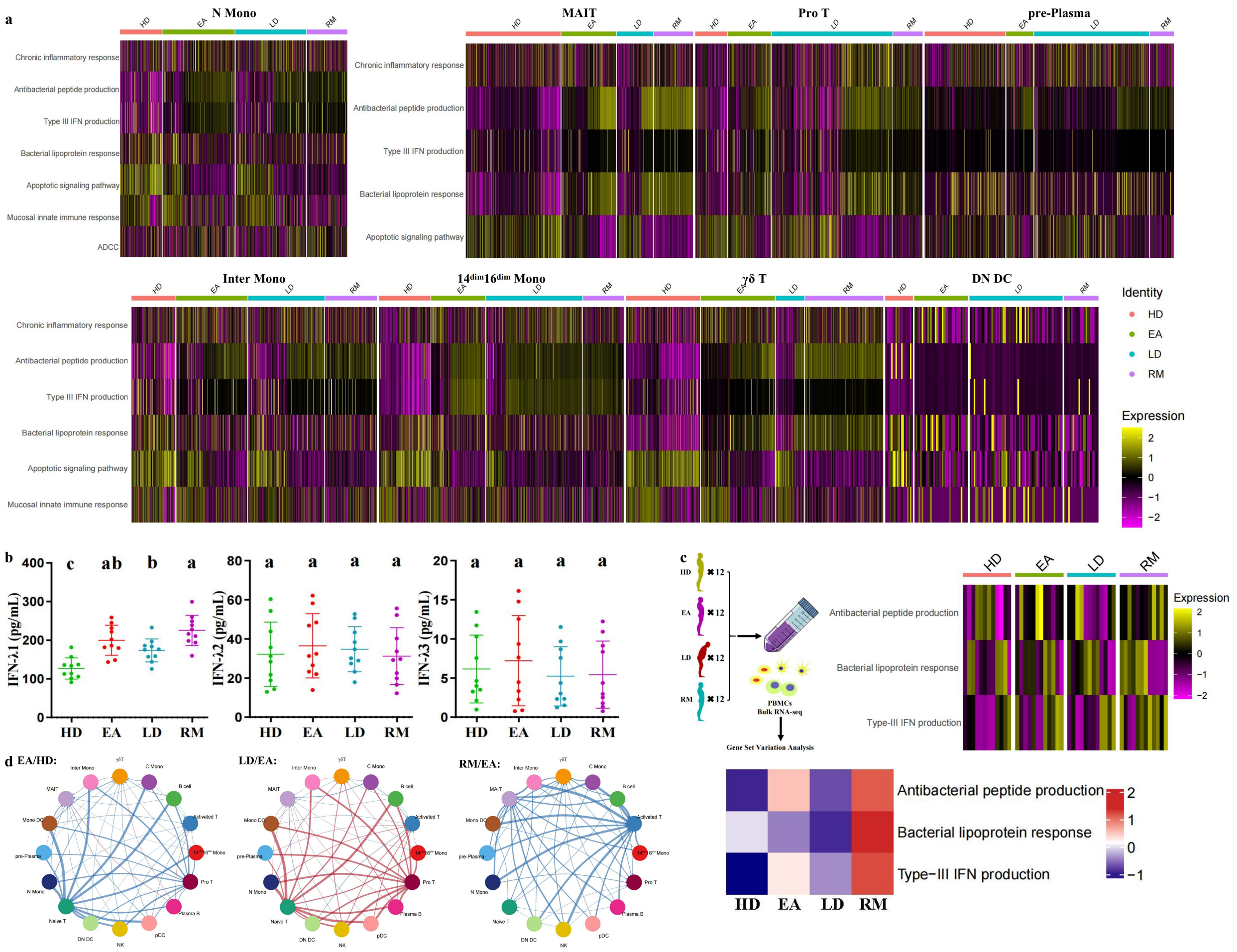

Supplementary Fig. 2 Functional comparisons of other cell types across AS conditions. **a** Heat maps showing relative intergroup comparisons of GSVA scores in multiple functions from corresponding cell types. Rows were normalized, and the transition of purple-black-yellow indicates an increase in the scores. **b** ELISA assays regarding serum levels of IFN-λ1/2/3 for each ten subjects in four groups. The demotion in letters indicates a significant decrease with $P<0.05$, and double-letter indicates no statistical difference between given group and compared groups. One-way ANOVA and Tukey Post-Hoc multiple comparisons. **c** PBMCs of each 12 subjects in the four groups were used for bulk RNA-Seq and the effective DEGs were prepared for GSVA. Relative intergroup comparisons of single-sample GSVA scores (top: the transition of purple-black-yellow indicates an increase in the scores) and overall GSVA scores (bottom: the transition of blue-white-red indicates an increase in the scores) for three innate-defense functions. **d** Intercellular communications showing differential number of actions from other cell types to 5 types of T cells. The changes were observed between corresponding groups.

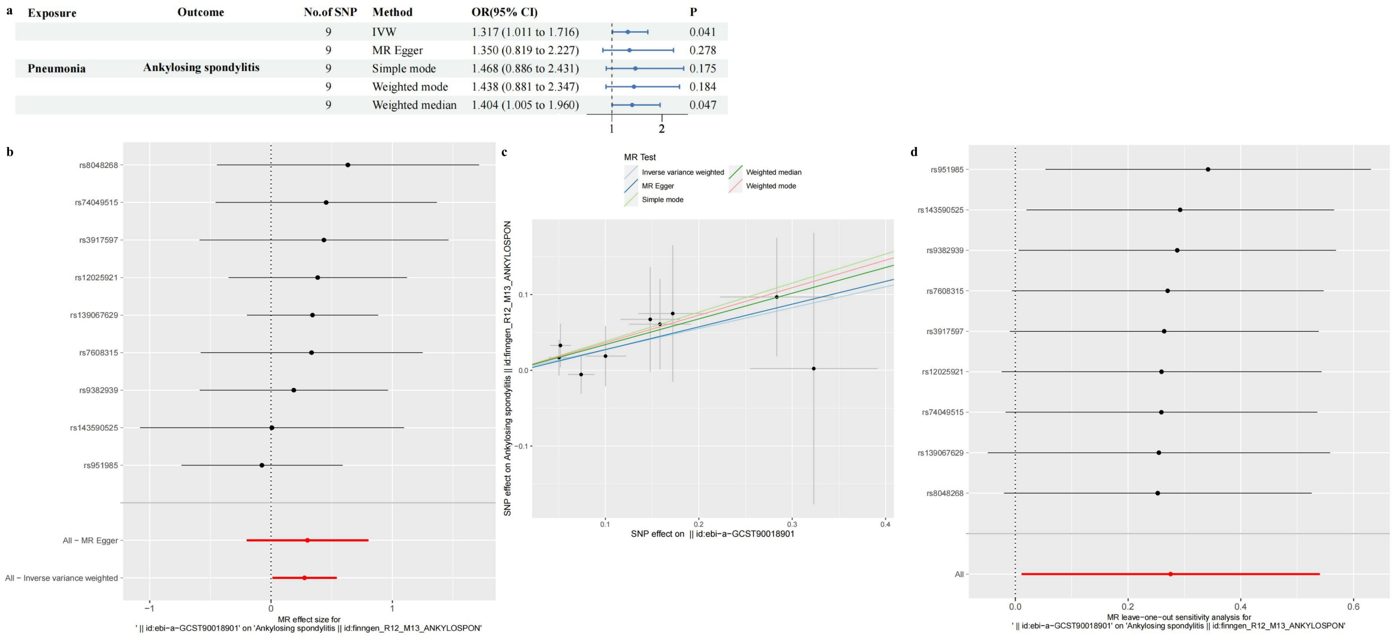

Supplementary Fig. 3 Mendelian randomization analysis of infection-AS causal relationship. a Forest plot of MR causality between pneumonia and AS. 14 single-nueleotide polymorphisms (SNPs) related to pulmonary infection were screened and after harmonisation with AS data, a total of 9 SNPs were included in the analysis. Inverse variance weighting [IVW; β=0.275, SE=0.135, OR=1.317, 95% CI (1.011, 1.716), P=0.041] indicated that infection is a risk factor for AS. **b** The average causal-estimated-values of IVW and MR-Egger analysis for 9 SNPs were over 0. The red lines represent the overall estimated values and 95% CI of IVW and MR-Egger analysis. **c** Linear regression analysis showed that the gradients of five MR tests were all over 0 and the trends of IVW and MR-Egger were similar, indicating that there is a positive correlation between pulmonary infection and AS and validating the effectiveness of instrumental variables. Horizontal axis represents the impact of SNPs on pneumonia, and vertical axis represents the impact of SNPs on AS. **d** The leaveone analysis showed that all SNPs were over 0, indicating that the effect values of SNPs fluctuated within a small range and MR causality remained stable.

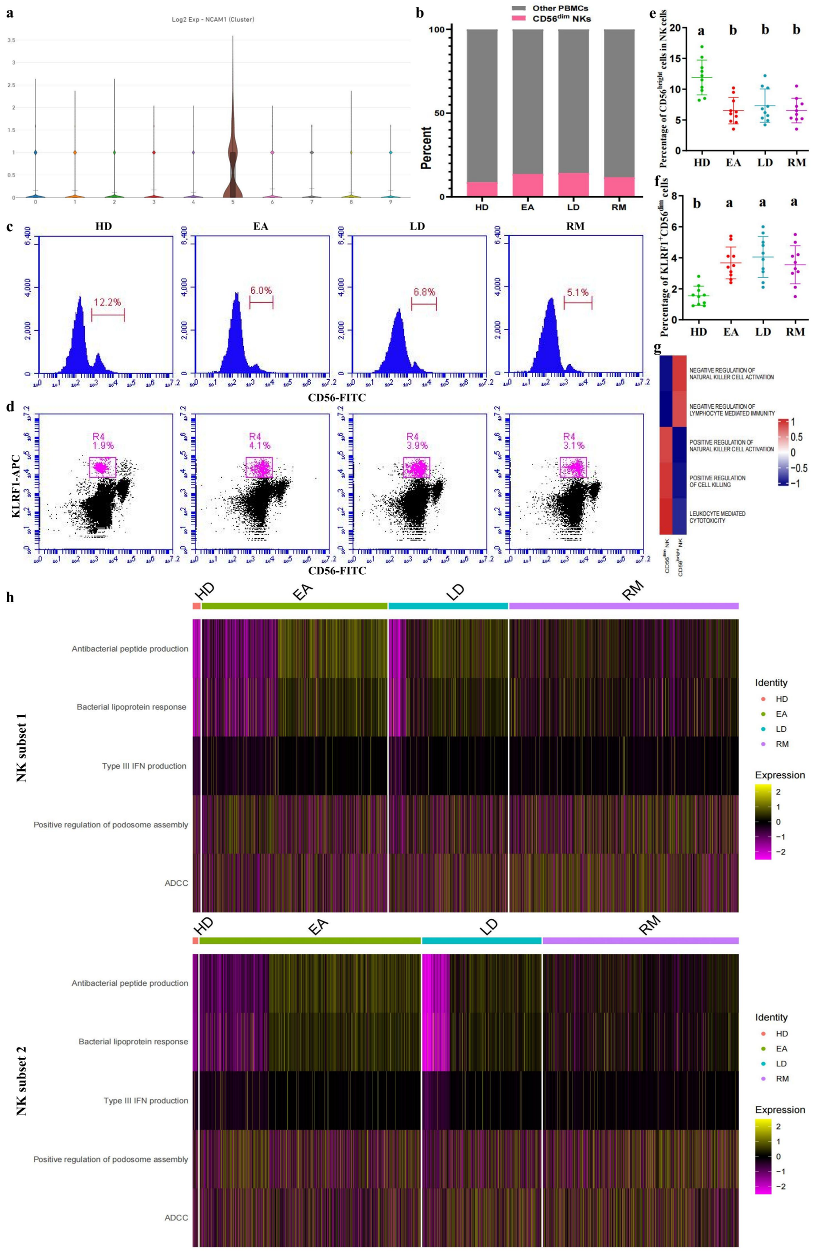

Supplementary Fig. 4 Identification of two NK subtypes and their dynamic features across AS conditions. a Violin chart showing the expression distribution of *CD56* (*NCAM1*) in 10 NK-cell subsets. **b** Proportions of *CD56*^{dim} NK-cells to total PBMCs across four groups. **c and e** Flow cytometry identifying the approximate trend of *CD56*^{bright} NK-cells from scRNA-seq. Data come from four groups of enriched NK-cells (sorting with KLRF1 antibody). **d and f** Flow cytometry showing the trend of KLRF1^{+}CD56^{dim} cells. Data come from PBMCs of each ten subjects in four groups. The demotion in letters indicates a significant decrease with $P<0.05$. One-way ANOVA and Tukey Post-Hoc multiple comparisons (**e,f**). **g** Relative comparisons of overall GSVA scores in multiple functions between *CD56*^{dim} NK-cells and CD56^{bright} NK-cells. Rows were normalized and clustered, and the transition of blue-white-red indicates an increase in the scores. **h** Relative comparisons of GSVA scores in multiple functions between four groups from NK subsets-1/2. Rows were normalized, and the transition of purple-black-yellow indicates an increase in the scores.

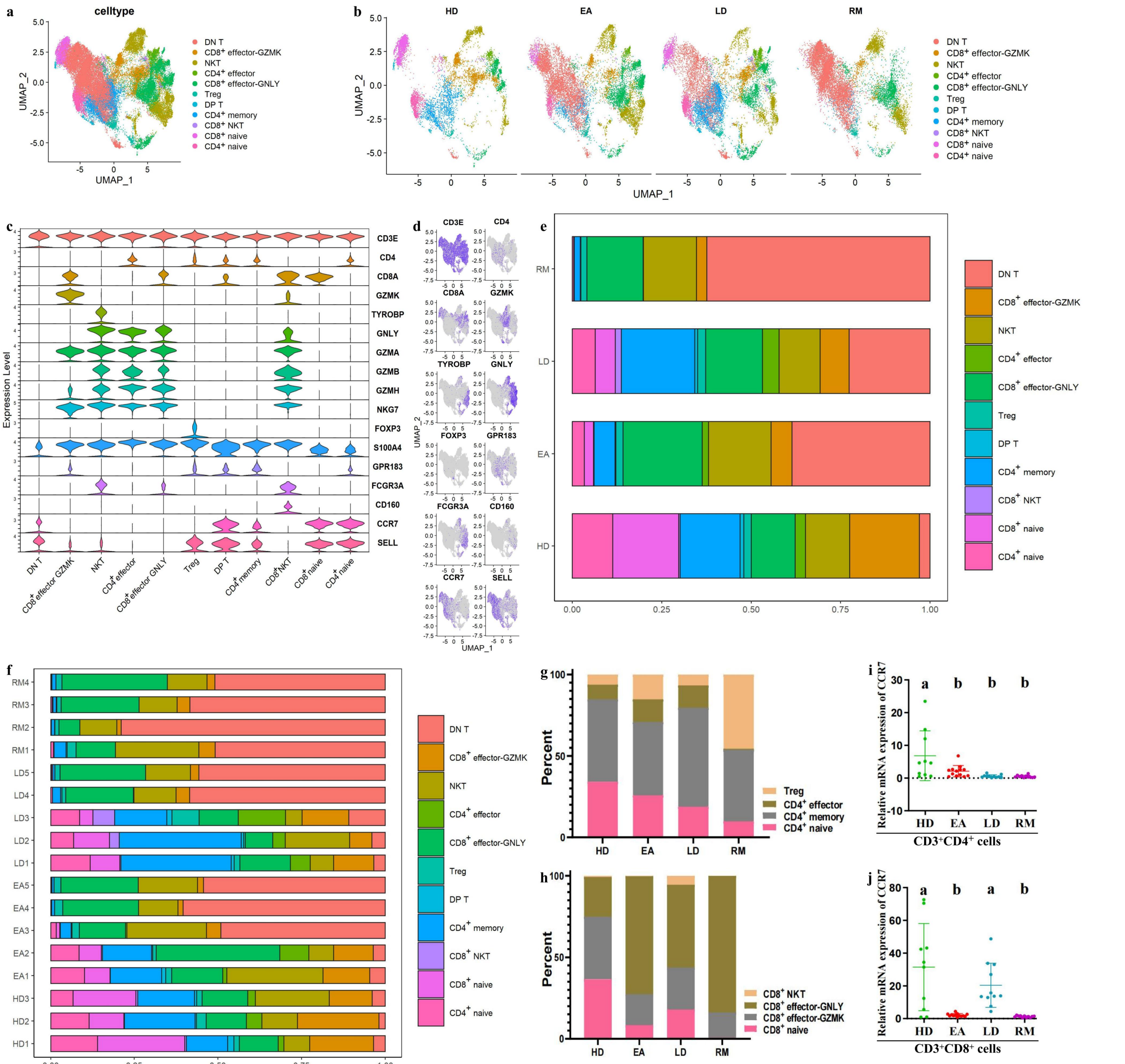

Supplementary Fig. 5 Characteristics of T-cell subtypes across AS conditions. a UMAP projection of 47,341 T cells. **b** T-cell UMAP projection of four conditions. **c** Violin chart showing the expression distribution of typical markers in 11 T-cell subtypes. **d** Typical cell markers in individual UMAP plots were colored according to the expression distribution. **a-d** $CD4^+$ T-cell subtypes included naive $CD4^+$ ($CD4^+$ naive) cells (*CCR7*$^+$*SELL*$^+$), memory $CD4^+$ ($CD4^+$ memory) cells (*S100A4*$^+$*GPR183*$^+$), effector $CD4^+$ ($CD4^+$ effector) cells (*GZMA*/*B*/*H*$^+$*GNLY*$^+$*NKG7*$^+$) and regulatory T (Treg) cells (*FOXP3*$^+$); $CD8^+$ T-cell subtypes included naive $CD8^+$ ($CD8^+$ naive) cells (*CCR7*$^+$*SELL*$^+$), $CD8^+$ effector-GZMK cells, $CD8^+$ effector-GNLY cells and $CD8^+$ NKT-cells (*CD8A*$^+$*FCGR3A*$^+$*CD160*$^+$); Other subtypes included double-negative NKT-cells (*CD4*$^-$*CD8A*$^-$*TYROBP*$^+$), double-negative T (DN T) cells (*CD4*$^-$*CD8A*$^-$) and double-positive T (DP T) cells (*CD4*$^+$*CD8A*$^+$). **e** Proportions of T-cell subtypes from four groups. **f** Proportions of 11 subtypes at sample levels. **g-h** Respective proportion of 4 $CD4^+$ T-cell subtypes or 4 $CD8^+$ T-cell subtypes to their total across four groups. **i-j** qPCR analysis of *CCR7* levels in enriched $CD4^+$ or $CD8^+$ T-cells (sorting with CD3-FITC and CD4-APC/CD8-PE antibodies) from four groups. The demotion in letters indicates a significant decrease with $P<0.05$. One-way ANOVA and Tukey Post-Hoc multiple comparisons.

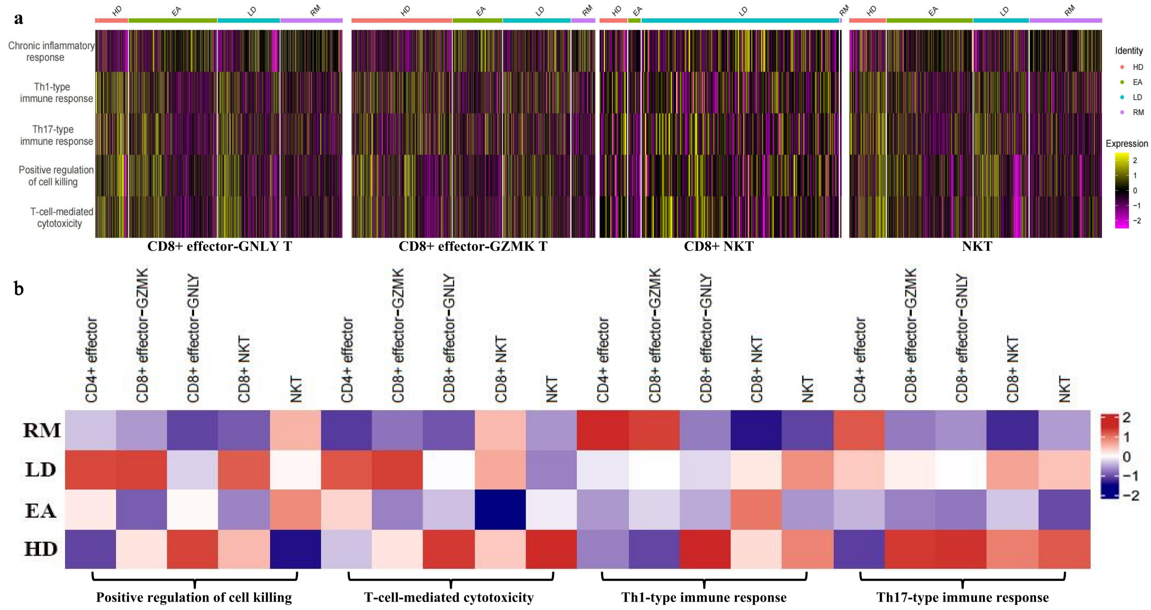

Supplementary Fig. 6 Functional comparisons of effector T-cell subtypes across AS conditions. a Relative intergroup comparisons of GSVA scores in multiple functions from CD8^{+} effector-GNLY, CD8^{+} effector-GZMK, CD8^{+} NKT and NKT cells. Rows were normalized, and the transition of purple-black-yellow indicates an increase in the scores. **b** Relative intergroup comparisons of overall GSVA scores for specific functions of various effector T-cell subtypes. Columns were normalized, and the transition of blue-white-red indicates an increase in the scores.

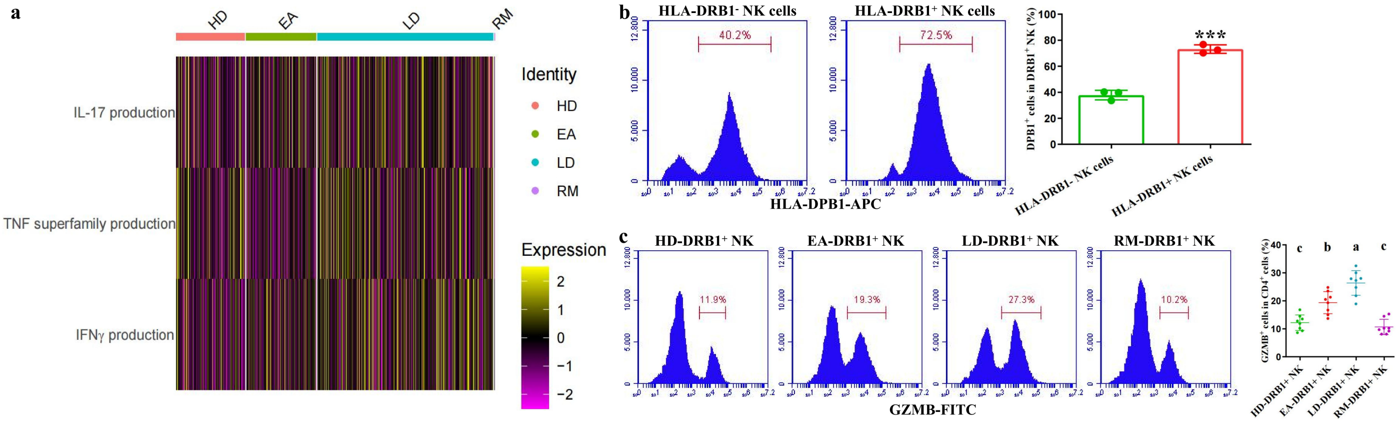

Supplementary Fig. 7 Supplementary data on the influence of APC-NK on CD4^{+} T-cell activation. a Relative comparisons of GSVA scores in the three representative cytokines responsible for AS lesions between groups from CD4^{+} effector T-cells. Rows were normalized, and the transition of purple-black-yellow indicates an increase in the scores. **b** The proportion of HLA-DPB1-positive cells in HLA-DRB1^{-} and HLA-DRB1^{+} NK-cells. ***P<0.001 by Student's t-tests. **c** The proportions of GZMB^{+} cells in the CD4^{+} T-cells cocultured with HLA-DRB1^{+} NK-cells from four gruops (n=8). The demotion in letters indicates a significant decrease with P<0.05. One-way ANOVA and Tukey Post-Hoc multiple comparisons.

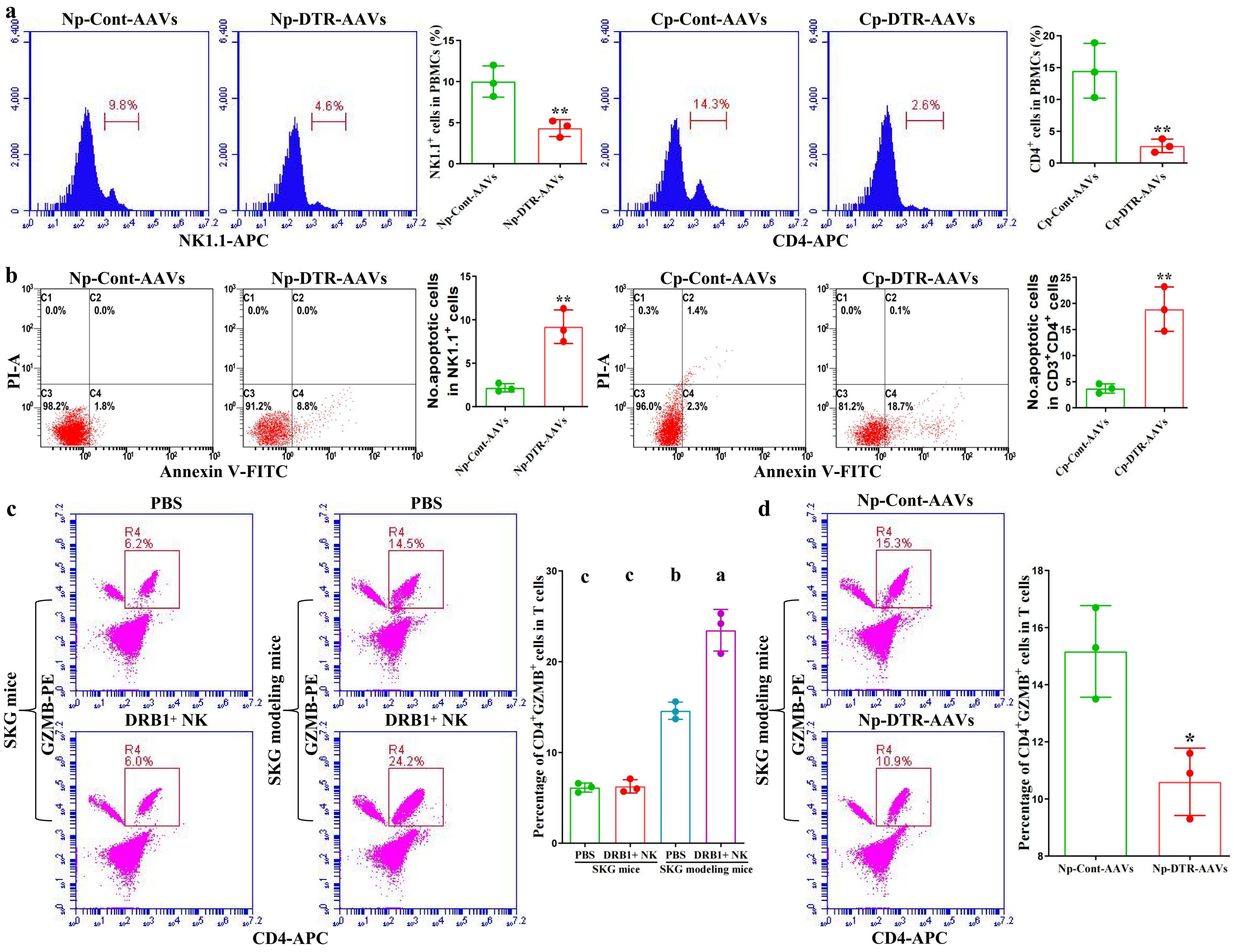

Supplementary Fig. 8 Supplementary data on the significance of NK cells/CD4 + T-cells for AS-like alterations. a Flow cytometry showing the proportions of NK1.1$^+$ cells or CD4$^+$ cells in PBMCs from each two groups of mice (n=3). **b** Flow cytometric analysis of apoptotic levels in enriched NK-cells (sorting with NK-1.1-APC antibody) and CD4$^+$ T-cells (sorting with CD3-PerCP/Cyanine5.5 and CD4-APC antibodies)(Annexin-V-positive cells represent apoptotic cells) from each two groups of mice (n=3). **c,d** Flow cytometry showing the proportions of CD4$^+$GZMB$^+$ T-cells in mouse T cells (sorting with CD3-PerCP/Cyanine5.5 antibody) for each group (n=3). The demotion in letters indicates a significant decrease with *P*<0.05. One-way ANOVA and Tukey Post-Hoc multiple comparisons. **P*<0.05, ***P*<0.01 by Student's t-tests.

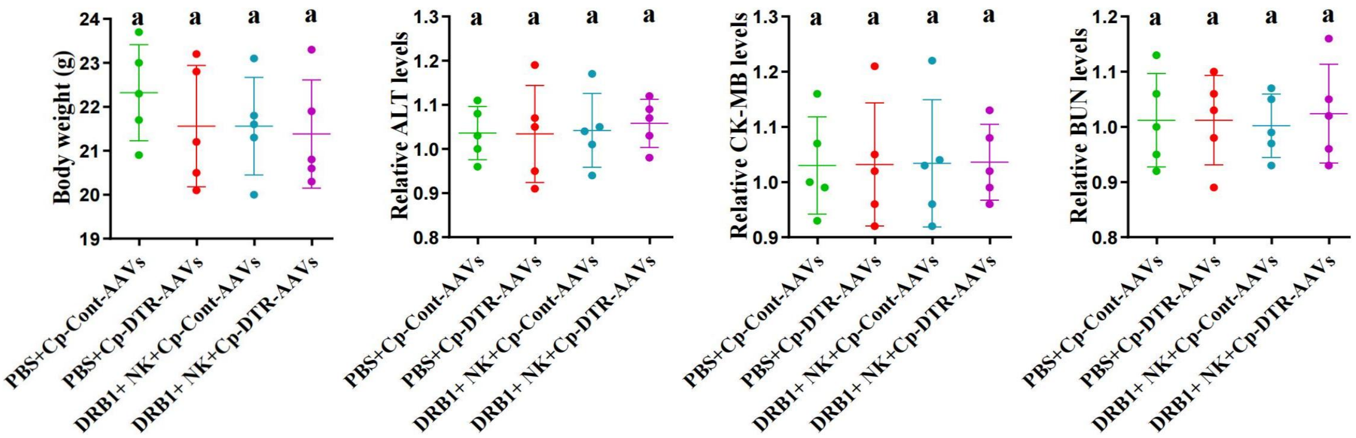

Supplementary Fig. 9 Quantification of body weight or serum ALT, CK-MB and BUN levels. The detection of serum ALT, CK-MB and BUN relies on clinical chemistry methods, and one sample from PBS+Cp-Cont-AAVs group was used as a control for normalization (n=5).

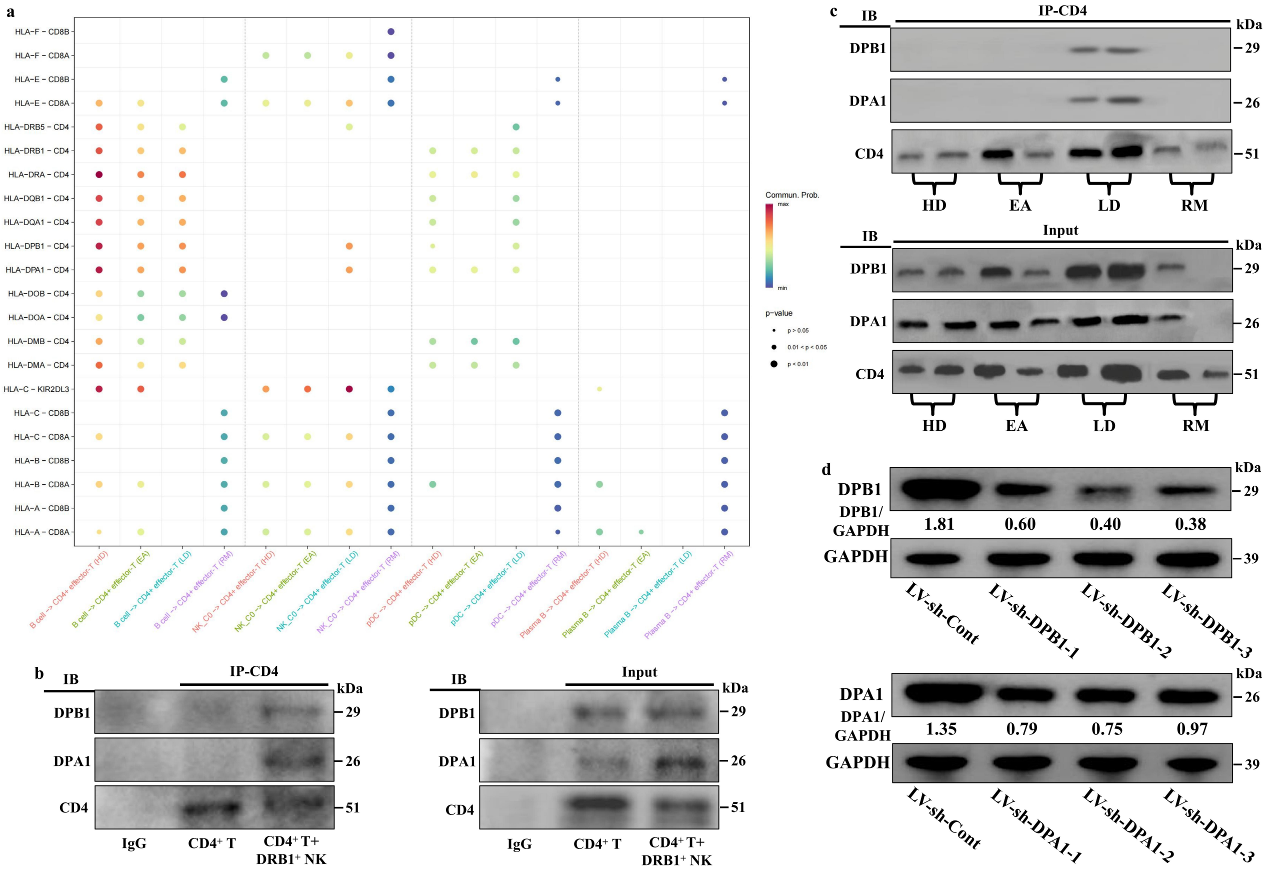

Supplementary Fig. 10 Intercellular interaction between HLA-DPB1/DPA1 in APC-NK and CD4 in CD4⁺ T-cells. a Intercellular communication analyses showing the communication probability between HLA genes in various APCs and surface receptors in CD4⁺ effector T-cells across four conditions. **b** COIP assays showing the interaction of CD4 and HLA-DPB1 or HLA-DPA1 in the cocultured cells including HLA-DRB1⁺ NK-cells and CD4⁺ T-cells. Data come from six independent samples with unanimous results from LDs. **c** COIP assays showing CD4—HLA-DPB1/HLA-DPA1 interactions in the cocultured cells from four groups. Data come from six independent samples with unanimous results. **d** According to Western-blot results, the silencing efficiency of LV-sh-DPB1-3 or LV-sh-DPA1-2 was considered the strongest. Accordingly, cells transduced by the optimal shRNAs were used for subsequent assays.

Supplementary Table 1 Characteristics of the subjects regarding scRNA-seq

	**HD**	**EA**	**LD**	**RM**
Number	3	5	5	4
Age, year	32.67±2.08	33.8±0.84	38.8±3.11	33.75±8.34
Number (%) male	3 (100%)	4 (80%)	4 (80%)	3 (75%)
Number (%) HLA-B27$^+$	3 (100%)	5 (100%)	5 (100%)	4 (100%)
Disease duration, month	NA	2.2±0.57[c]	89.6±26.79[a]	27.25±8.1[b]
Remission duration, month	NA	0[b]	0[b]	17.25±12.5[a]
CRP, mg/L	NA	4.66±2.82[b]	16.34±10.53[a]	0.81±0.1[c]
ESR, mm/h	NA	14±7.31[b]	35.8±19.42[a]	2.5±1.73[c]
BASDAI	NA	5.88±1.71[a]	5.12±1.8[a]	0.65±0.3[b]
BASFI	NA	5.16±2.35[a]	7.68±1.46[a]	1.35±0.53[b]

Mean±SD. HD, healthy donors; EA, patients in early-active stage; LD, patients in late-dysfunction stage; RM, patients in clinical remission; HLA-B27, human leukocyte antigen B27; CRP, C-reactive protein; ESR, Erythrocyte sedimentation rate; BASDAI, Bath ankylosing spondylitis disease activity index; BASFI, Bath ankylosing spondylitis functional index; NA, Not Applicable. The demotion in letters indicates a significant decrease with $P<0.05$.

Supplementary Table 2 Characteristics of the additional subjects for blood testing

	HD	EA	LD	RM
Number	9	8	7	8
Age, year	32.11±4.37	30±4.69	39.29±3.68	33.5±3.78
Number (%) male	7 (78%)	7 (88%)	6 (86%)	7 (88%)
Number (%) HLA-B27^{+}	0	8 (100%)	6 (100%)	7 (100%)
Disease duration, month	NA	2±0.65[c]	81.43±36.12[a]	31.63±16.89[b]
Remission duration, month	NA	0[b]	0[b]	19.13±14.75[a]
CRP, mg/L	NA	4.87±2.13[b]	12.48±3.08[a]	1.29±0.87[c]
ESR, mm/h	NA	13.88±2.17[b]	32.86±8.78[a]	6.38±3.85[c]
BASDAI	NA	5.61±1.62[a]	5.2±0.8[a]	0.79±0.29[b]
BASFI	NA	4.33±2.21[a]	7.23±1.65[a]	1.61±0.68[b]

Mean±SD. HD, healthy donors; EA, patients in early active stage; LD, patients in late dysfunction stage; RM, patients in clinical remission; HLA-B27, human leukocyte antigen B27; CRP, C-reactive protein; ESR, Erythrocyte sedimentation rate; BASDAI, Bath ankylosing spondylitis disease activity index; BASFI, Bath ankylosing spondylitis functional index; NA, Not Applicable. The demotion in letters indicates a significant decrease with $P<0.05$.

Supplementary Table 3 Characteristics of the subjects for ligament histological assessment

	Non-AS	AS
Number	4	5
Age, year	36.43±8.75	38.4±3.98
Number (%) male	4 (100%)	5 (100%)
Number (%) HLA-B27^{+}	0	5 (100%)
Disease duration, month	NA	68.4±37
CRP, mg/L	1.97±1.32	11.43±2.3
ESR, mm/h	5.25±2.5	33±9.67
BASDAI	NA	5.08±0.65
BASFI	NA	7.8±1.61

Mean±SD. AS, ankylosing spondylitis; HLA-B27, human leukocyte antigen B27; CRP, C-reactive protein; ESR, Erythrocyte sedimentation rate; BASDAI, Bath ankylosing spondylitis disease activity index; BASFI, Bath ankylosing spondylitis functional index; NA, Not Applicable.

Supplementary Table 4 Specific primer sequences for qRT-PCR

Gene	Forward（5'-3'）	Reverse（5'-3'）
CCR7	TGTGGTCGTGGTCTTCATAGTCTTC	CGTAGGCGATGTTGAGTTGCTTAC
HLA-DRB1	ACCGTGCTAATCCCTGAGTGTC	TGACCTGTGCTGATGGAGATGAG
HLA-DPB1	CCAGGAGAAGAGGCAGGTCAG	GGTGGCAGAGGATAGTGGAGATG
HLA-DPA1	GGTGGCAGAGGATAGTGGAGATG	CCAGGAGAAGAGGCAGGTCAG
GAPDH	GGAGCGAGATCCCTCCAAAAT	GGCTGTTGTCATACTTCTCATGG

Supplementary Table 5 MHC-II (DPB1/DPA1) binding prediction results for ovalbumin peptide

Alleles	Seq_start	Seq_end	Seq_length	Core_peptide	Peptide	%Rank
HLA-DPA1*01:03 /DPB1*18:01	127	141	15	LEPINFQTA	RGGLEPINFQTAADQ	0.01
HLA-DPA1*01:03 /DPB1*15:01	127	141	15	LEPINFQTA	RGGLEPINFQTAADQ	0.05
HLA-DPA1*01:03 /DPB1*08:01	127	141	15	LEPINFQTA	RGGLEPINFQTAADQ	0.09
HLA-DPA1*01:03 /DPB1*16:01	127	141	15	LEPINFQTA	RGGLEPINFQTAADQ	0.1
HLA-DPA1*01:03 /DPB1*02:01	127	141	15	LEPINFQTA	RGGLEPINFQTAADQ	0.1
HLA-DPA1*01:03 /DPB1*04:02	127	141	15	LEPINFQTA	RGGLEPINFQTAADQ	0.11
HLA-DPA1*01:03 /DPB1*02:02	127	141	15	LEPINFQTA	RGGLEPINFQTAADQ	0.11
HLA-DPA1*01:03 /DPB1*19:01	127	141	15	LEPINFQTA	RGGLEPINFQTAADQ	0.18
HLA-DPA1*01:03 /DPB1*01:01	127	141	15	LEPINFQTA	RGGLEPINFQTAADQ	0.26
HLA-DPA1*01:03 /DPB1*04:01	127	141	15	LEPINFQTA	RGGLEPINFQTAADQ	0.28

Supplementary Table 6 Representative labels and number of various cell type based on scRNA-seq

Cell type	Representative labels	Cell number
Activated T	$CD3^{+}PRF1^{+}$	16,491
Naive T	$CD3^{+}CCR7^{+}$	26,840
γδT	$TRGV9^{+}TRDV2^{+}$	1,789
MAIT	$SLC4A10^{+}TRAV1\text{-}2^{+}$	1,186
Pro T	$CD3^{+}MKI67^{+}$	1,035
NK	$KLRF1^{+}$	15,464
B	$MS4A1^{+}$	6,026
Plasma B	$MZB1^{+}$	1,233
pre-Plasma	$MZB1^{dim}IGLV3-25^{+}$	3,607
C Mono	$CD14^{bright}CD16^{dim}$	27,493
N Mono	$CD14^{dim}CD16^{bright}$	7,668
Inter Mono	$CD14^{bright}CD16^{bright}$	2,517
$14^{dim}16^{dim}$ Mono	$CD14^{dim}CD16^{dim}$	976
Mono DC	$CD1C^{+}$	2,426
pDC	$LILRA4^{+}$	1,041
DN DC	$CD1C^{-}THBD^{-}CSF3R^{bright}NEAT1^{bright}$	144
Mega	$PPBP^{+}$	2,177
HSC	$GATA2^{+}CYTL1^{+}$	221
